# Recent Advances in ZIF-8 Performance for Electrochemical Applications: A Comprehensive Review

**DOI:** 10.3390/ijms27177975

**Published:** 2026-09-07

**Authors:** Omirzak Abdirashev, Assem Temirbayeva, Gaukhar Kabdrakhimova, Balzhan Satanova, Aisulu Abuova, Fatima Abuova, Yerbol Ussen, Yerbolat Kalpakov, Marina Konuhova, Anatoli I. Popov

**Affiliations:** 1 Institute of Physical and Technical Sciences, L. N. Gumilyov Eurasian National University, Astana 010000, Kazakhstan; abdirashevomirzak@gmail.com (O.A.); assem.zkgmu@gmail.com (A.T.); satanova_bm_2@enu.kz (B.S.); abuova_au@enu.kz (A.A.); abuova_fu@enu.kz (F.A.); erbol_usen@mail.ru (Y.U.); 2 School of Intelligent Systems, Astana IT University, EXPO C1, Mangilik El 55/11, Astana 010000, Kazakhstan; yerbolat.kalpakov@alumni.nu.edu.kz; 3 Institute of Solid State Physics, University of Latvia, LV-1063 Riga, Latvia; popov@latnet.lv; 4 Ventspils International Radio Astronomy Centre, Ventspils University of Applied Sciences, LV-3601 Ventspils, Latvia

**Keywords:** electrochemical applications, hydrogen fuel cells, proton exchange membrane fuel cells, zeolitic imidazolate framework-8, ionic conductivity, chemical stability, catalytic activity, composite membranes

## Abstract

Zeolitic imidazolate framework-8 (ZIF-8) has emerged as a material for electrochemical energy conversion, serving dual primary roles in fuel cell technologies: (i) as an electrocatalyst precursor for oxygen reduction reaction (ORR) and methanol oxidation reaction (MOR) through pyrolysis-derived N-doped porous carbons and metal–nitrogen–carbon (M–N–C) structures, and (ii) as a membrane component that enhances proton conductivity via imidazole-mediated Grotthuss hopping while suppressing fuel crossover through molecular sieving. This comprehensive review systematically evaluates ZIF-8 performance across multiple fuel cell types, including primarily proton exchange membrane fuel cells (PEMFCs), as well as direct methanol fuel cells (DMFCs), anion exchange membrane fuel cells (AEMFCs), and microbial fuel cells (MFCs), while also covering related electrochemical applications such as zinc–air batteries, supercapacitors, and water splitting devices, where ZIF-8-derived materials demonstrate improved catalytic activity. The review examines structure–performance relationships, highlighting strategies such as heteroatom doping, bimetallic synergy, hierarchical porosity engineering, and polymer composite fabrication that have enabled ZIF-8-based catalysts to achieve ORR half-wave potentials and PEMFC power densities, rivaling commercial Pt/C systems. ZIF-8 composite membranes demonstrate proton conductivities in polybenzimidazole systems and effective methanol blocking. Despite improved progress, challenges persist regarding long-term stability, scalable synthesis, and degradation mechanism understanding. This review critically analyzes recent advances, identifies performance-limiting factors across applications, and outlines future research directions for developing commercially viable ZIF-8-based electrochemical technologies.

## 1. Introduction

### 1.1. Scope and Rationale: ZIF-8 for Electrochemical Applications with Emphasis on PEMFCs

**Figure 1:**
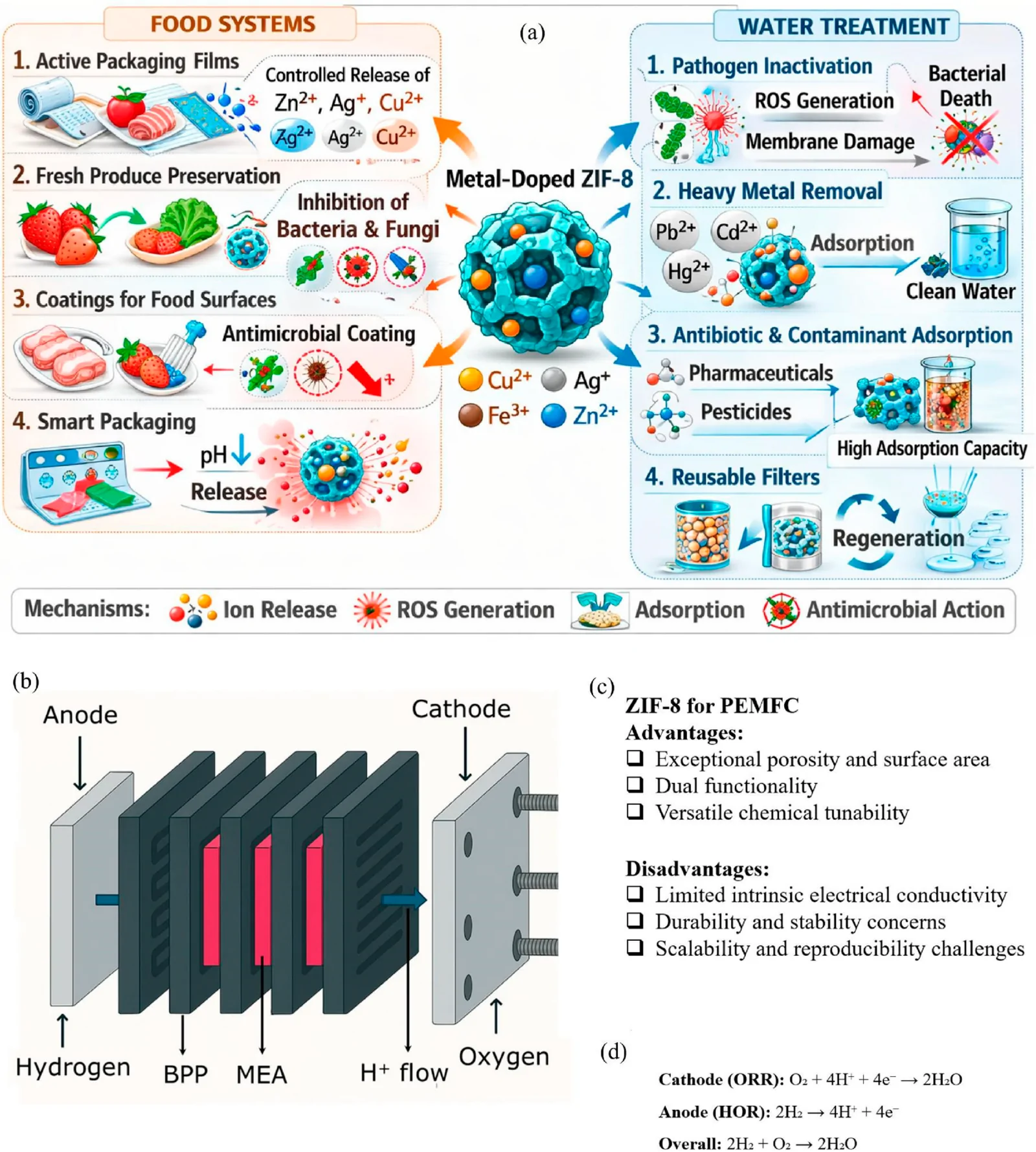
(**a**) The utilization of ZIF-8 and its modified counterparts, specifically silver, copper, and iron-functionalized ZIF-8, within the contexts of food safety and aqueous environments. Reprinted from (CC BY license) [3]. (**b**) Schematic representation of a PEMFC stack. Reprinted from (CC BY license) [4]. (**c**) Advantages and disadvantages of ZIF-8 for PEMFCs. (**d**) Cathodic, anodic, and overall reactions in a PEMFC.

The urgent global need for sustainable and clean energy technologies has positioned electrochemical energy conversion and storage systems as cornerstones of the future energy landscape. Among these, proton exchange membrane fuel cells (PEMFCs) offer high efficiency, low emissions, and potential applications in transportation, portable devices, and stationary power generation, converting chemical energy directly into electricity through electrochemical reactions (Figure 1) [1,2]. However, the widespread commercialization of PEMFCs is significantly hindered by two primary challenges: the sluggish kinetics of the oxygen reduction reaction (ORR) at the cathode, which requires substantial amounts of expensive and scarce platinum-based catalysts, and the limitations of current proton exchange membranes, particularly regarding performance at elevated temperatures and resistance to fuel crossover [3,4].

In response to these challenges, the exploration of advanced materials has become paramount, and among these, zeolitic imidazolate framework-8 (ZIF-8), a subclass of metal–organic frameworks (MOFs), has emerged as an important candidate. Structurally, ZIF-8 consists of zinc ions tetrahedrally coordinated by 2-methylimidazolate linkers, forming a sodalite-type zeolitic topology [5,6]. This structure bestows upon ZIF-8 a specific surface area, tunable porosity, thermal and chemical stability (up to 550 °C in inert atmospheres), and nitrogen-rich imidazole linkers that provide sites for proton hopping, heteroatom doping, and anchoring of catalytic metal nanoparticles [6,7,8].

Importantly, the versatility of ZIF-8 has motivated its investigation across a broader spectrum of electrochemical devices beyond conventional H_2_/O_2_ PEMFCs, including direct methanol fuel cells (DMFCs), anion exchange membrane fuel cells (AEMFCs), microbial fuel cells (MFCs), zinc–air batteries, supercapacitors, and water-splitting systems [9,10,11,12]. While the present review focuses principally on PEMFC applications, these adjacent technologies are included selectively for two reasons: (i) they share fundamental materials science principles with PEMFCs, particularly the use of ZIF-8-derived N-doped carbons, M–N–C active sites, and proton-conductive or ion-conductive membranes, and (ii) insights from these systems can inform and accelerate PEMFC-relevant developments. However, it must be emphasized that DMFCs, AEMFCs, MFCs, and other electrochemical devices differ fundamentally in their operating principles, reaction mechanisms, fuel types, and performance metrics; therefore, direct cross-device comparisons are avoided unless a scientifically valid basis exists.

Within the PEMFC domain specifically, research on ZIF-8 has branched into two principal high-impact directions. The first leverages ZIF-8 as an important precursor for synthesizing non-precious metal catalysts, addressing the critical need to reduce reliance on platinum. Through pyrolysis, ZIF-8 can be transformed into nitrogen-doped porous carbons (N–C) and metal–nitrogen–carbon (M–N–C) structures, where atomically dispersed transition metals (e.g., Fe, Co, or Mn) serve as highly active ORR sites [5,6,7]. Fe–N–C catalysts derived from ZIF-8 have demonstrated maximum power densities exceeding 1 W cm^−2^, rivaling state-of-the-art platinum-based systems. The second direction focuses on ZIF-8’s potential as a membrane component: its rigid microporous structure (pore aperture ~0.34 nm) can act as a molecular sieve to block fuel crossover, while its imidazole groups facilitate proton transport through Grotthuss-type hopping mechanisms, particularly when integrated with polymers such as Nafion or polybenzimidazole (PBI) [6,7,8,9,10]. Recent advances have even explored encapsulating proton carriers like deep eutectic solvents within ZIF-8 nanochannels, achieving high proton conductivity at elevated temperatures [9,10].

This review addresses the central question: “How do ZIF-8 and ZIF-8-derived materials function as both electrocatalysts (and catalyst precursors) and membrane components to enhance the performance, durability, and economic viability of proton exchange membrane fuel cells and related electrochemical energy conversion devices?”. While this overarching question encompasses three interconnected sub-questions, concerning membrane function, catalyst function, and applications across devices, they are unified by a common materials platform and are systematically examined within a single integrated framework.

Despite the extensive literature on ZIF-8 for electrochemical applications, several critical knowledge gaps remain. First, there is no unified framework that systematically distinguishes among the different material forms of ZIF-8, pristine framework, chemically functionalized ZIF-8, polymer composites, metal-decorated structures, and pyrolyzed carbonaceous derivatives, and clarifies how each contributes to performance through distinct mechanisms. Second, the literature lacks a systematic analysis of whether performance improvements arise from intrinsic ZIF-8 properties (porosity, imidazole functionality), polymer–ZIF-8 interfacial effects (acid–base interactions, hydrogen bonding), guest species (water, ionic liquids, phosphoric acid), structural defects (surface Zn-OH sites), incorporated metal ions (Fe, Co, Ni, Cu), or transformations during pyrolysis (formation of M–N_x_ active sites, graphitization). Third, no comprehensive comparison exists of how these different material forms perform across various electrochemical devices (PEMFCs, DMFCs, AEMFCs, MFCs, zinc–air batteries, supercapacitors, water splitting) with respect to application-specific performance metrics and degradation mechanisms [9,10,11,12,13,14,15].

To address these gaps, this review develops an integrated framework that connects material structure, function, testing conditions, and device performance. This enables readers to understand not only what has been achieved but also why certain material designs succeed or fail in specific applications. By systematically categorizing ZIF-8 materials by form and function, and by critically analyzing the underlying mechanisms of performance enhancement, this review provides a roadmap for the rational design of ZIF-8-based materials for next-generation electrochemical energy conversion technologies.

Compared with previously published reviews, which typically focus exclusively on either MOF-based membranes, MOF-derived catalysts, or ZIF-8 for a single application, this review provides a unique integrated perspective by systematically examining both the membrane and catalyst functions of ZIF-8 within a unified framework. While existing reviews have comprehensively covered MOF materials for fuel cells [3,6,9] or ZIF-8 synthesis and properties [11,14], few have critically compared the distinct requirements, performance metrics, and degradation mechanisms of membrane versus catalyst applications using the same material platform. This review adds substantive value beyond the existing literature by: (i) developing a systematic classification of ZIF-8 material forms (pristine, functionalized, composite, derived) and their respective performance-enhancing mechanisms; (ii) critically analyzing whether reported improvements arise from intrinsic ZIF-8 properties, interfacial effects, guest species, defects, metal ions, or pyrolysis transformations; (iii) comparing performance across multiple electrochemical devices with explicit recognition of different operating conditions and metrics; and (iv) identifying which conclusions are supported by multiple independent studies versus isolated claims requiring further validation. By integrating these analyses, this review bridges the gap between fundamental materials science and practical electrochemical device performance, offering a clear roadmap for future research.

### 1.2. Methodology of Literature Survey

To ensure a systematic, transparent, and reproducible review process, we employed a structured methodology including (i) literature search strategy, (ii) inclusion and exclusion criteria, and (iii) study selection, data extraction, and categorization.

We conducted comprehensive searches across multiple databases including Web of Science, Scopus, Google Scholar, and PubMed. The search covered publications from January 2006 to June 2026 to capture both foundational work on ZIF-8 synthesis and properties as well as recent advances in electrochemical applications. The search strategy combined keywords related to ZIF-8 materials with application-specific terms using Boolean operators (AND, OR, NOT) (Table 1).

Studies were included if they met all of the following criteria: (i) peer-reviewed original research articles, reviews, or communications published in English; (ii) focused on ZIF-8 or ZIF-8-derived materials; (iii) directly related to electrochemical energy conversion applications (fuel cells, batteries, supercapacitors, water splitting, CO_2_ reduction); and (iv) reported quantitative performance data (conductivity, power density, current density, overpotential, etc.). Studies were excluded if they: (i) focused exclusively on non-electrochemical applications (drug delivery, gas adsorption, photocatalysis, sensing without electrochemical relevance); (ii) lacked sufficient experimental detail to evaluate performance; (iii) were conference abstracts, book chapters, or preprints without peer review; or (iv) were not available in English (Table 2).

Data extracted from each study included: synthesis method and conditions, material characterization results (surface area, porosity, morphology, composition), application type and testing conditions (temperature, pressure, pH, feed gases), performance metrics (conductivity, power density, current density, half-wave potential, durability), and reported mechanistic insights. Studies were categorized by: (i) material form (pristine ZIF-8, functionalized ZIF-8, ZIF-8/polymer composites, ZIF-8/metal composites, ZIF-8-derived carbons, ZIF-8-derived M–N–C catalysts); (ii) application type (PEMFCs, DMFCs, AEMFCs, MFCs, zinc–air batteries, supercapacitors, water splitting, other); and (iii) material function (catalyst, membrane, or both). This categorization enabled systematic comparison within application types while respecting the distinct operating conditions and performance metrics of each device. Performance data were extracted directly from tables and figures, with reported values converted to consistent units where possible. When comparing results, we explicitly noted differences in experimental conditions (e.g., H_2_/O_2_ vs. H_2_/air, acidic vs. alkaline electrolyte, temperature, pressure, loading) and avoided direct cross-device comparisons without appropriate contextualization.

### 1.3. Scope and Organization of This Review

**Figure 2:**
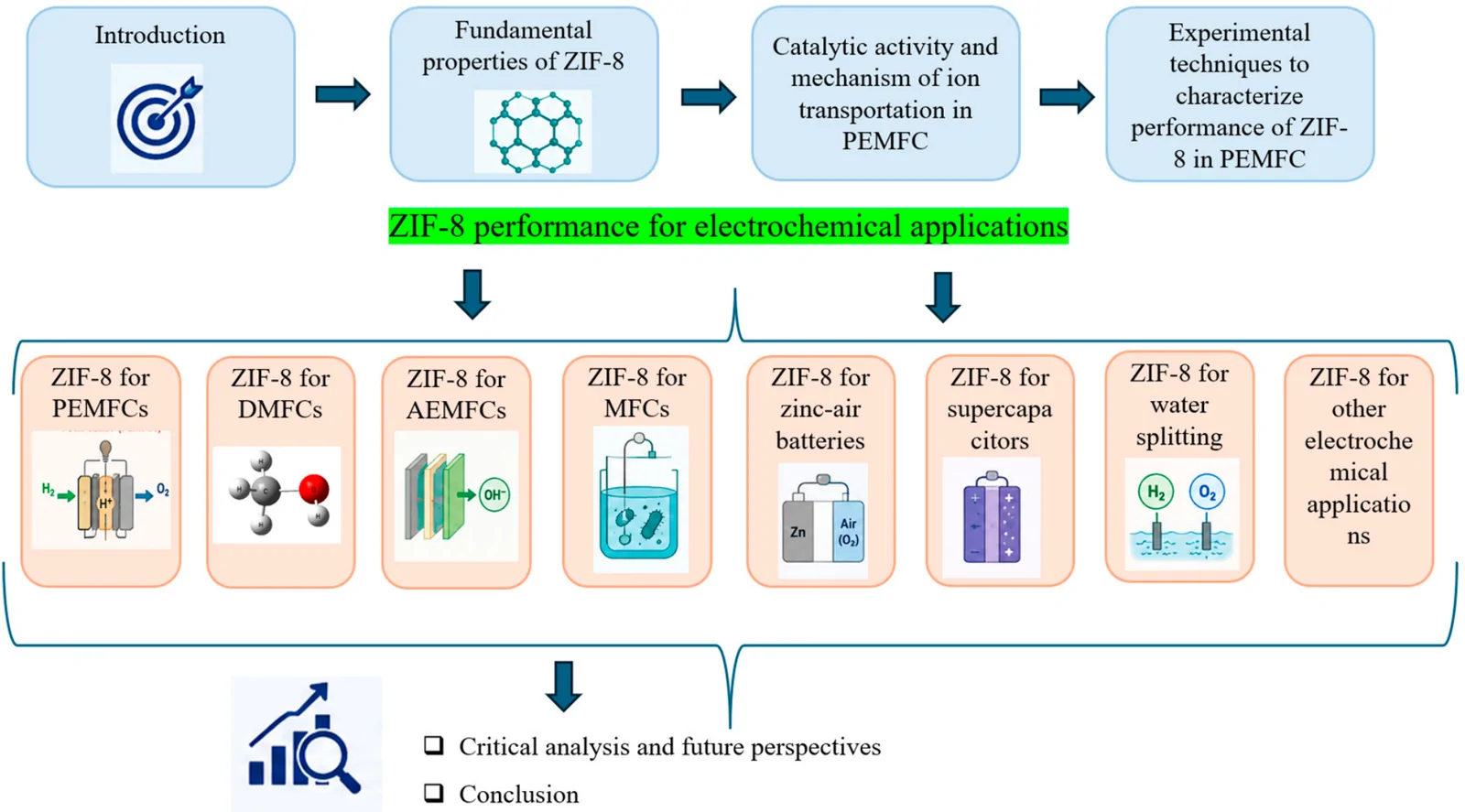
Schematic overview of the organization and main contents of this review.

This comprehensive review provides a systematic examination of ZIF-8’s multifunctional roles across a wide spectrum of electrochemical energy conversion and storage systems. While PEMFCs represent a primary application area, the versatility of ZIF-8 and its derived materials has enabled their deployment in numerous other electrochemical technologies, including DMFCs, AEMFCs, MFCs, zinc–air batteries, supercapacitors, water splitting systems, and other electrochemical devices (Figure 2).

The scope encompasses pristine ZIF-8, ZIF-8-derived materials (N-doped porous carbons, metal–nitrogen–carbon structures, and composite systems), and ZIF-8-based composite, with particular emphasis on structure–performance relationships across various device architectures. The review is organized as follows: Section 2 presents the fundamental properties of ZIF-8, including crystal structure, porosity, morphology, and chemical/thermal stability, establishing the materials science foundation essential for understanding its diverse applications. Section 3 elucidates the catalytic activity and mechanisms of ion transport through ZIF-8 frameworks, covering proton conduction pathways, molecular sieving effects, and charge transport principles applicable across multiple electrochemical systems. Section 4 surveys the experimental characterization techniques employed to evaluate ZIF-8 performance across different applications. Section 5 provides comprehensive performance analysis systematically organized into application-specific subsections including PEMFCs, DMFCs, AEMFCs, MFCs, zinc–air batteries, supercapacitors, water splitting, and other emerging applications. While the primary focus of this review is on electrochemical energy conversion devices, Section 5.8.7 discusses hydrogen storage as a related enabling technology. Although hydrogen storage is not an electrochemical application in the strict sense, it is included here because onboard hydrogen storage remains a critical barrier to PEMFC commercialization, and ZIF-8-based materials have shown promise for this application. This inclusion provides context for the PEMFC community and highlights the broader relevance of ZIF-8 to the hydrogen economy. Section 6 offers critical analysis of current challenges including stability limitations, scalability issues, degradation mechanisms, and cross-cutting challenges common to all applications, while outlining future research directions. Section 7 presents concluding remarks on the prospects of ZIF-8-based materials for practical electrochemical technologies and their role in sustainable energy conversion.

## 2. Fundamental Properties of ZIF-8

### 2.1. Crystal Structure and Porosity

ZIF-8 crystallizes in a cubic space group $I\bar{4}3m$ with a sodalite (SOD) topology, where each Zn^2+^ ion is tetrahedrally coordinated by four 2-methylimidazolate (Hmim) linkers. This arrangement creates a three-dimensional porous network with alternating large cavities (~11.6 Å diameter) and narrow windows (~3.4 Å aperture), forming a cage-like structure with the Zn···Zn distance of approximately 6.0 Å. The rigid framework exhibits crystallinity with lattice parameter a ≈ 16.99 Å, confirmed by powder X-ray diffraction (PXRD) showing characteristic peaks at 2θ = 7.3°, 10.4°, 12.7°, and 18.0°, corresponding to (011), (002), (112), and (222) planes, respectively [9,10,11,12,13].

The porosity of ZIF-8 is improved, with typical Brunauer–Emmett–Teller (BET) surface areas ranging from 1400 to 1800 m^2^/g and total pore volumes of 0.6–0.8 cm^3^/g. These values depend strongly on synthesis conditions, with solvothermal methods generally yielding higher surface areas than room-temperature routes. The microporous nature (pore size < 2 nm) dominates, though hierarchical porosity can be achieved through template-assisted synthesis or controlled structural transformation [10,11,12,13,14]. Nitrogen adsorption–desorption isotherms typically exhibit Type I behavior characteristic of microporous materials. The high surface area provides abundant sites for metal nanoparticle anchoring, heteroatom doping, and reactant accessibility, directly influencing catalytic and transport performance in fuel cell applications.

### 2.2. Morphology

ZIF-8 exhibits improved morphological diversity that profoundly influences its functional properties. The most common morphology is rhombic dodecahedral crystals with sizes ranging from 50 nm to several micrometers, which can be systematically controlled by varying synthesis parameters such as reaction temperature, precursor concentration, and solvent composition. Nanocrystalline ZIF-8 (sub-100 nm) possesses higher concentrations of Zn–Im_2_ surface defects, providing additional hydrophilic sites that enhance water-mediated proton transport, with conductivity values two orders of magnitude higher than micron-sized powders.

Beyond conventional dodecahedra, ZIF-8 can adopt various architectures including nano-sized crystallites (~52 nm), rectangular plates, hollow structures, nanosheets, and core–shell configurations. The hollow structures, generated through template-assisted or etching strategies, offer enhanced mass transport and active site accessibility. Two-dimensional ZIF-8 nanosheets present maximized surface area and shortened diffusion pathways, particularly beneficial for membrane applications [13,14,15,16]. Morphological engineering through surfactant-assisted synthesis, solvent modulation, and epitaxial growth enables precise control over particle size, shape, and hierarchical organization. These morphological features critically affect ion conductivity, catalytic activity, and compatibility with polymer matrices, making morphology control an essential consideration in ZIF-8-based fuel cell applications.

### 2.3. Chemical and Thermal Stability

The chemical stability of ZIF-8 is improved among MOFs, primarily attributed to the strong coordination bonds between Zn^2+^ and the 2-methylimidazolate linkers. ZIF-8 demonstrates improved resistance to water, organic solvents, and mild acidic/basic conditions. In aqueous environments, the hydrophobic nature of the methylimidazolate linkers protects the framework from hydrolysis, maintaining structural integrity over extended periods. However, prolonged exposure to strongly acidic or basic solutions can induce framework degradation through protonation of imidazolate linkers or zinc hydroxide formation. The stability under operating fuel cell conditions is particularly critical, as the membrane environment involves humidified gases at elevated temperatures.

Thermally, ZIF-8 exhibits stability, with its framework remaining intact up to approximately 550 °C in inert atmospheres. Thermogravimetric analysis (TGA) reveals minimal weight loss below 400 °C, attributed to removal of solvent molecules, with framework decomposition initiating around 500–550 °C through organic linker combustion [14,15,16,17,18]. The thermal stability correlates with particle size, where nanocrystalline ZIF-8 shows slightly reduced decomposition temperatures. In oxidative environments, decomposition occurs at lower temperatures due to organic combustion. This thermal robustness enables high-temperature pyrolysis (600–1000 °C) for converting ZIF-8 into nitrogen-doped porous carbons while preserving the sodalite-derived framework structure. The combined chemical and thermal stability positions ZIF-8 as a platform for both membrane and catalyst applications in PEMFCs operating under challenging conditions (Table 3).

## 3. Mechanism of Ion Transport and Catalytic Activity in PEMFC

The improved ion transport and catalytic properties of ZIF-8 in PEMFCs arise from a complex interplay of structural features, surface chemistry, and external stimuli. Proton conduction through ZIF-8-based systems operates primarily through two complementary mechanisms: the Grotthuss mechanism (hopping) and the vehicle mechanism, both enabled by the framework’s unique architecture and functional groups [19,20].

The Grotthuss mechanism dominates proton transport in hydrated ZIF-8 systems, where protons hop between adjacent imidazole linkers and water molecules through hydrogen-bonded networks. The nitrogen atoms in the 2-methylimidazolate linkers serve as proton donors and acceptors, facilitating rapid proton transfer with relatively low activation energies. This hopping mechanism is particularly efficient when hydrophilic guest molecules (e.g., water, imidazole, phosphoric acid) are confined within ZIF-8’s hydrophobic nanochannels, creating continuous hydrogen-bonding pathways [18,19,20,21,22]. The vehicle mechanism operates synergistically, where proton carriers such as water molecules, imidazole, or phosphoric acid diffuse through ZIF-8’s interconnected pores, transporting protons via molecular motion. The rigid microporous structure (3.4 Å aperture) provides well-defined pathways while simultaneously acting as a molecular sieve [19,20,21,22,23,24]. This molecular sieving effect is particularly advantageous for direct methanol fuel cells, as the pore aperture (~3.4 Å) effectively blocks methanol molecules (~3.8 Å), reducing fuel crossover by orders of magnitude compared to commercial Nafion membranes [23,24].

Composite systems further enhance ion transport through synergistic effects. When ZIF-8 is incorporated into polymer matrices, acid–base pairs between ZIF-8’s imidazole groups and the polymers’ sulfonic acid or phosphoric acid groups reduce the energy barrier for proton transfer. For instance, Nafion/ZIF-8@PCM composites achieve proton conductivity of 0.24 S cm^−1^ at 80 °C, while PBI/ZIF-8 membranes demonstrate conductivities up to 0.316 S cm^−1^ at 160 °C [25,26,27,28]. The confined water molecules and functional groups within ZIF-8 nanopores create efficient long-range proton transport highways, making ZIF-8 an important platform for high-performance PEMFC electrolytes.

Beyond ion transport, ZIF-8 demonstrates significant catalytic activity, primarily through its derived carbonaceous materials. Pristine ZIF-8 exhibits limited intrinsic ORR activity due to its insulating nature and lack of metal active sites [5,6,29,30,31,32,33,34,35]. However, upon pyrolysis (600–1000 °C), ZIF-8 transforms into nitrogen-doped porous carbons with atomically dispersed M–N_x_ active sites (M = Fe, Co, Mn), where transition metals coordinate with pyridinic nitrogen to form catalytic centers [7,8,36,37,38,39,40,41,42,43,44,45,46,47]. These M–N_x_ sites facilitate the four-electron ORR pathway by optimizing O_2_ adsorption and *OH desorption energetics, achieving half-wave potentials approaching 0.9 V in acidic media [48,49,50,51,52,53,54,55,56,57,58,59,60,61]. The catalytic activity is further enhanced through heteroatom doping (N, S, B, P), bimetallic synergy (Fe–Co, Fe–Ni), and hierarchical porosity engineering that improves mass transport and active site accessibility [43,44,60]. The combination of proton-conducting capabilities in pristine form and improved catalytic activity upon pyrolysis establishes ZIF-8 as an important material platform for PEMFC applications, addressing both membrane and catalyst challenges simultaneously.

## 4. Experimental Techniques to Characterize Performance of ZIF-8 in PEMFC

Comprehensive characterization of ZIF-8 performance in electrochemical applications requires a multi-technique approach spanning structural, morphological, electrochemical, and device-specific analyses. These techniques provide crucial insights into material properties, ion transport mechanisms, catalytic activity, and device performance across various applications.

Regarding structural and morphological characterization, powder X-ray diffraction (PXRD) is essential for confirming ZIF-8 crystallinity and phase purity, with characteristic peaks at 2θ = 7.3°, 10.4°, 12.7°, and 18.0° corresponding to the sodalite topology. Scanning electron microscopy (SEM) and transmission electron microscopy (TEM) reveal particle morphology, size distribution, and crystal habit, while high-resolution TEM provides atomic-scale structural information [25,26,27,28,29]. Brunauer–Emmett–Teller (BET) surface area analysis via nitrogen physisorption at 77 K quantifies porosity, with ZIF-8 typically exhibiting Type I isotherms characteristic of microporous materials. Thermogravimetric analysis (TGA) coupled with differential scanning calorimetry (DSC) evaluates thermal stability and framework decomposition temperatures [14,15,16,17,18].

Regarding membrane-specific characterization, electrochemical impedance spectroscopy (EIS) is the primary technique for determining proton conductivity, typically performed using two-probe or four-probe configurations across a frequency range of 1 Hz to 1 MHz. Through-plane conductivity measurements utilize membrane samples sandwiched between electrodes in humidity-controlled chambers, while in-plane measurements employ interdigitated electrodes. Conductivity is calculated from the Nyquist plot [27,28,29,30,31,32]. Temperature (25–180 °C) and relative humidity (0–100% RH) dependencies reveal activation energy via Arrhenius plots, elucidating proton transport mechanisms (Grotthuss vs. vehicle). Water uptake and swelling ratio measurements assess dimensional stability. Methanol permeability is determined using diffusion cell setups with gas chromatography or refractive index monitoring, evaluating fuel crossover resistance [32,33,34,35,36]. Mechanical properties (tensile strength, Young’s modulus) are characterized via stress–strain testing. Ion exchange capacity (IEC) is measured through titration, and oxidative stability is assessed by Fenton’s test.

Regarding catalyst-specific characterization, rotating disk electrode (RDE) and rotating ring-disk electrode (RRDE) techniques evaluate ORR activity in liquid electrolytes (0.1 M HClO_4_ or KOH). These measurements provide onset potential, half-wave potential (E_1_/_2_), electron transfer number (n), and hydrogen peroxide yield, critical indicators of catalyst activity and selectivity. Cyclic voltammetry (CV) assesses electrochemical surface area, while linear sweep voltammetry (LSV) provides polarization curves [30,31,32,33]. For membrane electrode assemblies (MEAs), single-cell testing in fuel cell hardware generates polarization curves (voltage vs. current density) and power density plots, measuring actual device performance under H_2_/O_2_ or H_2_/air feeds with specified operating conditions [32,33,34,35,36].

Regarding surface and chemical analysis, X-ray photoelectron spectroscopy (XPS) identifies surface chemical states, nitrogen configurations (pyridinic-N, pyrrolic-N, graphitic-N, M–Nx), and metal oxidation states. Raman spectroscopy characterizes carbon defect densitY, while Fourier-transform infrared spectroscopy (FTIR) confirms functional groups and chemical interactions [27,28,29,30,31,32,33,34]. X-ray absorption spectroscopy (XAS), including XANES and EXAFS, provides coordination environment and local structure information for metal sites, crucial for confirming atomically dispersed M–Nx active centers.

Regarding application-specific testing, for fuel cell applications (PEMFCs, DMFCs, AEMFCs), single-cell testing evaluates polarization curves, power density, and durability under relevant operating conditions (temperature, pressure, fuel type). For MFCs, electrochemical measurements are performed under neutral pH conditions with actual wastewater or synthetic substrates [41,42,43,44,45,46,47,48,49,50,51,52]. For zinc–air batteries, galvanostatic discharge and charge–discharge cycling tests evaluate specific capacity and cycling stability. Supercapacitors require galvanostatic charge–discharge (GCD), CV, and EIS measurements to determine specific capacitance and rate capability. Water splitting applications utilize linear sweep voltammetry and chronoamperometry to assess HER and OER overpotentials and Tafel slopes. For each application, the characterization techniques are tailored to the specific performance metrics and operating conditions, and results should be reported with complete experimental parameters to enable meaningful comparison within each application category.

## 5. Performance of ZIF-8

The diverse functionality of ZIF-8 for electrochemical applications is best understood through a systematic examination of its performance across various roles, ranging from pristine frameworks to derived catalysts and composite membranes. This section provides a comprehensive analysis of ZIF-8 performance organized by application type, with dedicated subsections for each electrochemical device category. Within each application, we critically evaluate material design strategies, structure–performance relationships, and key performance metrics while distinguishing between membrane and catalytic functions where applicable. For PEMFCs, the dual role of ZIF-8 is addressed in separate subsections, membrane applications and catalytic applications, to reflect the fundamentally different experimental methodologies, performance indicators, and degradation mechanisms associated with each function. Subsequent sections cover DMFCs, AEMFCs, MFCs, zinc–air batteries, supercapacitors, water splitting (HER/OER), and other emerging electrochemical applications. This organizational framework enables readers to access application-specific information while facilitating within-category comparative analysis, avoiding inappropriate cross-device performance comparisons. Each subsection synthesizes findings from multiple studies, identifies trends in material design and performance optimization, highlights knowledge gaps, and discusses application-specific challenges and future directions. The performance metrics, testing conditions, and benchmark materials are contextualized within each application’s unique operating principles and requirements.

### 5.1. ZIF-8 for Proton Exchange Membrane Fuel Cells (PEMFCs)

#### 5.1.1. Membrane Applications

ZIF-8 has emerged as an important filler material for polymer electrolyte membranes in PEMFCs, leveraging its rigid microporous structure (3.4 Å pore aperture), nitrogen-rich imidazole linkers, and excellent chemical stability to enhance proton conductivity while suppressing fuel crossover. The incorporation of ZIF-8 into polymer matrices such as Nafion, polybenzimidazole (PBI), and polyvinyl alcohol (PVA) creates acid–base pairs between ZIF-8’s imidazole groups and the polymers’ sulfonic acid or phosphoric acid groups, reducing the energy barrier for proton transfer through Grotthuss-type hopping mechanisms [2,19,20,21,22]. This section critically analyzes ZIF-8-based composite membranes across low-temperature (Nafion-based) and high-temperature (PBI-based) PEMFC applications, with emphasis on proton conductivity, stability, and the trade-offs between performance enhancement and mechanical integrity.

**Figure 3:**
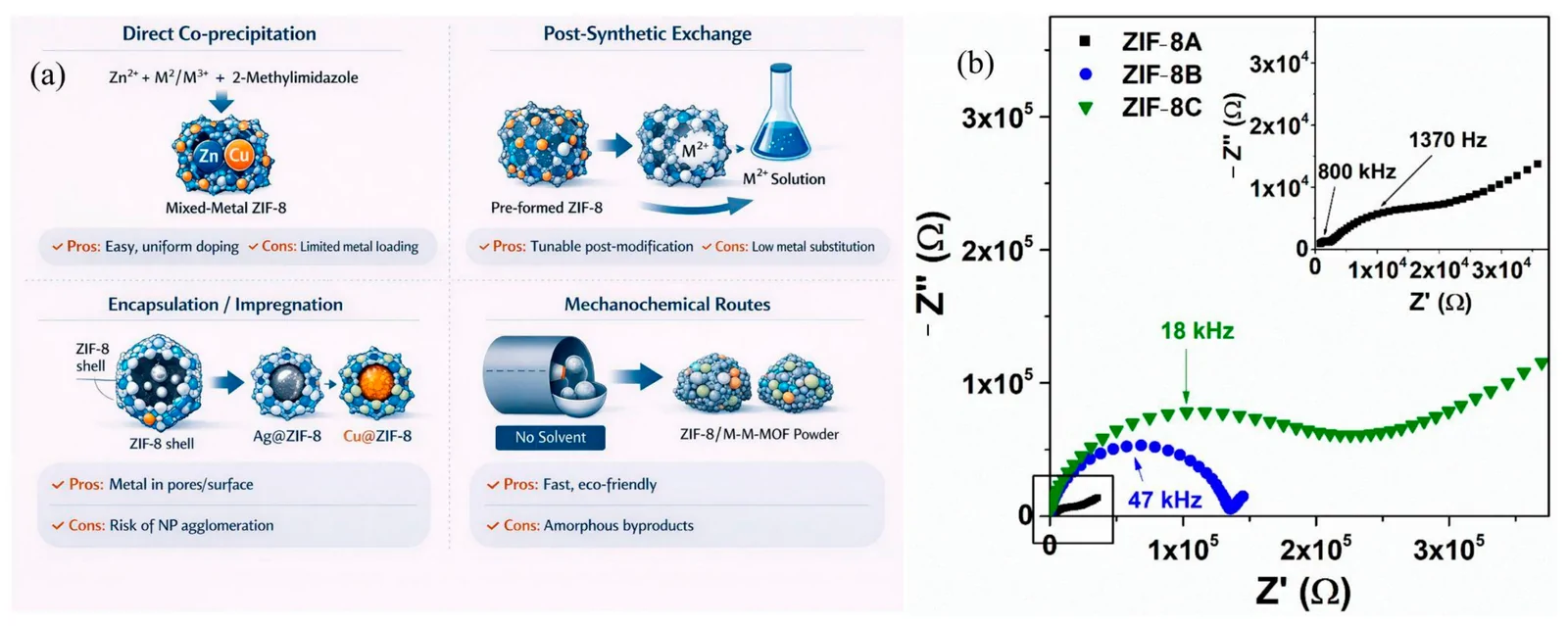
(**a**) Metal-doped ZIF-8 nanomaterials and its synthetic routes. Reprinted from (CC BY license) [3]. (**b**) Nyquist measurements were performed on ZIF-8A (black squares), ZIF-8B (blue circles), and ZIF-8C (green triangles) at 94 °C under a P/P_0_ of 0.98. Reprinted from (CC BY license) [2].

Muñoz-Gil et al. study demonstrates that nanostructuring ZIF-8 enhances proton conductivity through water-mediated surface transport. ZIF-8A (80 nm) exhibited conductivity of 0.5 mS/cm at 94 °C and 98% RH, two orders higher than micron-sized powders (Figure 3). XPS revealed that nanocrystalline ZIF-8 has a higher concentration of Zn–Im_2_ surface defects, providing hydrophilic sites for water adsorption. The surface water enables a different proton-conducting mechanism with lower activation energy (1.14 eV vs. 1.5 eV). This nanostructuring approach offers an alternative to chemical functionalization, which often compromises MOF stability. The strategy is generalizable to other MOFs for enhancing ionic conductivity [2].

Regarding Nafion-based composite membranes, Zhu et al. developed a novel 3D core–shell architecture where ZIF-8 nanoparticles were anchored on polyacrylate carboxyl microspheres (ZIF-8@PCM) and incorporated into Nafion [37]. The Nafion/ZIF-8@PCM-1 composite achieved proton conductivity of 0.24 S cm^−1^ at 80 °C and 100% RH, representing a 1.5-fold enhancement over recast Nafion (0.165 S cm^−1^). The 3D spherical fillers provided excellent dispersibility within the Nafion matrix, addressing the common challenge of nanoparticle agglomeration. The activation energy decreased from 10.96 kJ mol^−1^ for recast Nafion to 8.02 kJ mol^−1^ for the composite, confirming that acid–base pairs between N-H groups and -SO_3_H groups effectively reduced the energy barrier for proton transport. Similarly, Deng et al. developed a confined PFSA/ZIF-8 composite membrane by growing ZIF-8 on polydopamine-modified perforated stainless steel meshes, achieving maximum power density of 385 mW cm^−2^ at 60 °C—three times higher than Nafion 117 [38]. The confined architecture created an array of PFSA micropillars with high surface area-to-volume ratio (54,000 m^−1^), enabling polymer chain restructuring for enhanced proton transport.

Regarding high-temperature PEMFCs (HT-PEMFCs) operating above 100 °C, PBI membranes doped with phosphoric acid are preferred. Eren et al. fabricated PBI/ZIF-8 composite membranes with varying MOF loadings (2.5–10 wt%) [39]. The composite with 10 wt% ZIF-8 achieved proton conductivity of 0.316 S cm^−1^ at 160 °C, more than three-fold higher than pristine PBI (0.083 S cm^−1^). This enhancement was attributed to the Grotthuss and vehicle mechanisms facilitated by ZIF-8’s porous structure, providing additional pathways for proton hopping and phosphoric acid carrier molecules. However, decreased mechanical stability and increased hydrogen permeability were observed at higher loadings due to particle agglomeration. Devrim and Colpan further optimized PBI/ZIF-8 composites, reporting maximum power density of 432 mW cm^−2^ at 170 °C and proton conductivity of 0.163 S cm^−1^ with 2 wt% ZIF-8 loading [40]. Activation energy decreased from 19.78 kJ mol^−1^ for pristine PBI to 14.97 kJ mol^−1^ for the composite, confirming reduced proton transport barriers, while acid retention capability was significantly improved with minimal phosphoric acid leaching.

Across these studies, several consistent trends emerge. First, optimal ZIF-8 loading typically falls between 2 and 10 wt%, depending on the polymer matrix—exceeding this threshold leads to agglomeration and compromised mechanical properties. Second, the proton conductivity enhancement arises from a combination of intrinsic ZIF-8 properties (imidazole-mediated hopping) and interfacial effects (acid–base interactions), not simply from increased water uptake (Table 4). Third, the trade-off between conductivity and mechanical stability remains a critical challenge, with all studies reporting some degree of mechanical degradation at higher loadings.

#### 5.1.2. Catalytic Applications

ZIF-8 has emerged as an improved precursor for synthesizing non-precious metal electrocatalysts for PEMFCs, addressing the critical challenge of replacing expensive platinum-based catalysts for the ORR at the cathode. Through high-temperature pyrolysis (600–1100 °C), ZIF-8 transforms into nitrogen-doped porous carbons with atomically dispersed metal–nitrogen–carbon (M–N–C) active sites, where transition metals (Fe, Co, Mn, Zr) coordinate with pyridinic nitrogen to form catalytic centers [5,6,7]. This section critically analyzes ZIF-8-derived catalysts for PEMFC applications, focusing on active-site engineering, synthesis strategies, performance metrics, and the persistent challenge of durability (Table 5).

Regarding active-site engineering and design strategies, the catalytic activity of ZIF-8-derived materials originates primarily from atomically dispersed M–N_x_ sites, particularly Fe–N_4_ and Co–N_4_ configurations, which facilitate the four-electron ORR pathway by optimizing O_2_ adsorption and *OH desorption energetics [42,62]. Huang et al. demonstrated a multi-heteroatom-doped porous carbon catalyst using a 3D network of ZIF-8/polymeric nanofiber as a facile-doping template, achieving excellent ORR activity comparable to commercial 20% Pt/C with better durability and methanol crossover tolerance [41]. Liu et al. demonstrated that ultra-small nano-ZIF-8 (8 nm units) derived catalysts preferentially host edge-type FeN_4_ sites with doubled intrinsic activity, achieving 48.5 mA cm^−2^ at 0.9 V_iR-free and P_max of 1.41 W cm^−2^, meeting DOE 2025 targets [62]. The first quantitative active site density measurement using nitrite stripping (40 μmol g^−1^) provided crucial insights for catalyst design. Similarly, solid-phase synthesis of Fe-doped ZIF-8 (Fe_2_-Z8-C) yielded 3 wt% atomically dispersed Fe-N_4_ moieties without Fe nanoparticle formation, delivering current density of 1.65 A cm^−2^ at 0.6 V and P_max of 1.14 W cm^−2^ [42]. Zhao et al. demonstrated that Fe-ZIF precursors with Zn-ZIF supports yield excellent ORR activity with volumetric current density of 12 A cm^−3^ at 0.8 V, ranking among top non-PGM catalysts [63].

Heteroatom doping strategies have significantly enhanced ORR activity by modulating electron density and creating additional active sites. Ma et al. demonstrated Fe, B, and N co-doped hollow mesoporous carbon (Fe-BN-C-20) with E_1_/_2_ of 0.859 V, surpassing commercial Pt/C (0.853 V), with improved stability (91% retention after 36,000 s) [43]. The synergistic effect of multiple heteroatoms creates larger asymmetrical spin and higher charge density, improving ORR kinetics. Bimetallic approaches, such as Fe–Co, Fe–Ni, and Fe–Mn combinations, further enhance active site density and durability through synergistic electronic effects [44]. Zr co-doping with Fe (Fe/Zr-N-C) achieved improved durability with 40% retention after 100 h, compared to >70% loss for Fe-only catalysts within 20 h, attributed to Zr’s improved acid tolerance and suppression of H_2_O_2_ formation [45,46].

Regarding synthesis strategy and carbon architecture, the pyrolysis temperature critically influences active site formation, graphitization, and catalytic performance. Temperatures above 800 °C are essential for Fe–N–C formation, with higher temperatures improving chemical stability and electron conductivity [47]. However, excessive temperature can lead to metal agglomeration and active site loss. Lai et al. compared Joule heating (JH) versus furnace heating (FH) for ZIF-8 carbonization, finding that JH produces catalysts with higher graphitization but lower N content, yet exhibits higher intrinsic activity (TOF = 0.023 s^−1^ vs. 0.009 s^−1^) due to enhanced graphitization [48]. DFT confirmed graphitic-N sites have a lower energy barrier (0.27 eV) for OH* → H_2_O conversion, explaining improved performance.

Hierarchical porosity engineering has emerged as a critical strategy for overcoming mass transport limitations inherent in microporous ZIF-8-derived carbons. Qiao et al. constructed hierarchically ordered macro/meso/microporous carbon with atomically dispersed FeN_4_ using polystyrene sphere templates, achieving E_1_/_2_ of 0.80 V in 0.5 M H_2_SO_4_, only 20 mV inferior to Pt/C [47]. The hierarchical porous structure significantly enhanced mass transfer and active site accessibility compared to microporous FeN_4_/C. Zhao et al. developed dual-size architecture (100 nm “seeds” assembled by 30 nm “satellites”) with a hierarchically porous structure, achieving ORR E_1_/_2_ of 0.86 V and peak power density of 0.75 W cm^−2^, with only 24.5 mV loss after 30,000 cycles [49]. Carbon nanotube incorporation via seeded growth and MWCNT composites further improves interparticle conductivity and mass transport, with ZIF’-FA-CNT-p achieving 820 mW cm^−2^ through enhanced oxygen mass transport (limiting current density of 5531 mA cm^−2^) [50].

Regarding Co–N–C and bimetallic systems, Co–N–C catalysts derived from ZIF-67@ZIF-8 core–shell structures have demonstrated ORR activity, particularly when combined with conductive supports. Wang et al. synthesized Co–N–C via in situ growth on KJ600 carbon black, achieving improved P_max of 0.92 W cm^−2^ in H_2_-O_2_ PEMFC, comparable to state-of-the-art Fe–N–C catalysts [51]. The KJ600 support improved mass transfer and conductivity, reducing high-frequency resistance by 25 mΩ cm^−2^. The carboxylate-assisted approach by Li et al. boosted ORR mass activity of ZIF-derived Fe/N/C catalysts by 2–10 fold, achieving 5.98 A g^−1^ at 0.85 V and P_max of 1.33 W cm^−2^ in H_2_-O_2_ PEMFC—a improved for non-Pt catalysts—though stability remained poor (80.3% loss in 35 h) [52]. Seeded growth of branched Fe–N-doped CNTs using Fe-ZIF-8 precursor achieved 480 mW cm^−2^ with only 34% loss after 30 h AST, compared to 78% for Fe-ZIF catalyst [53]. Mooste et al. combined polymer-derived ceramics with ZIF-8 functionalization for HT-PEMFC cathodes, with CoFe-N-SiOCa exhibiting improved durability (ΔE1/2 = 8 mV after ADT) and HT-PEMFC performance (P100 = 34 mW cm^−2^, OCV = 768 mV) [54]. Sulfurization of core–shell ZIF-67@ZIF-8 further enhanced ORR kinetics in acidic media through increased defective sites and Co–N_x_ active sites [64], while core–shell ZIF-67@ZIF-8-derived multi-dimensional carbon (Co@N-CNT-HC) achieved half-wave potential of 0.84 V and improved durability (97.8% current retention after 8 h), attributed to synergistic effects of Co nanoparticles and N-doped carbon [65].

**Figure 4:**
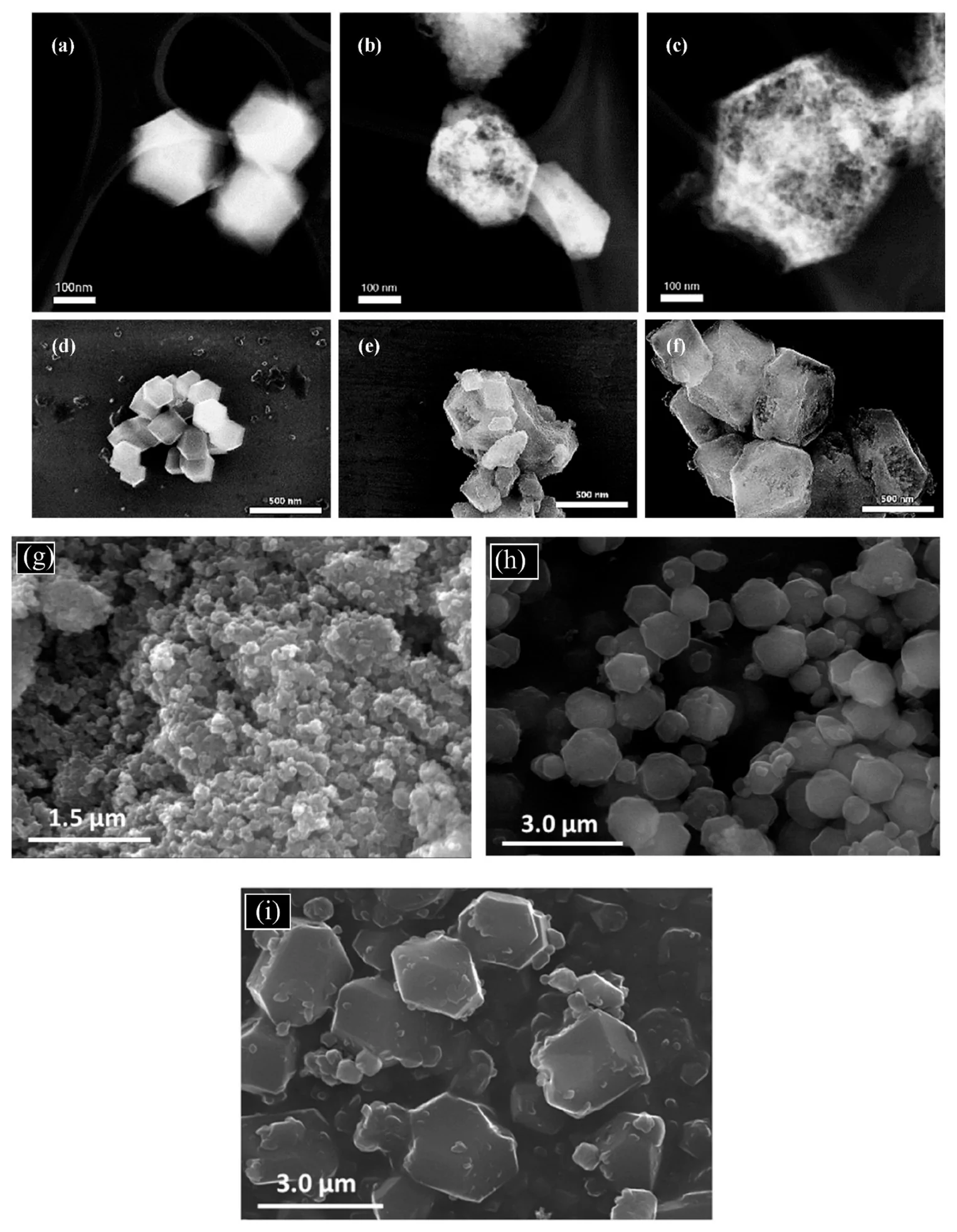
STEM images of (**a**) Meso-ZIF-NC-1, (**b**) Meso-ZIF-NC-5, and (**c**) Meso-ZIF-NC-6; SEM images of (**d**) Meso-ZIF-NC-1, (**e**) Meso-ZIF-NC-5, and (**f**) Meso-ZIF-NC-6. Reprinted from (CC BY license) [59]. SEM images of (**g**) ZIF-8A, (**h**) ZIF-8B, and (**i**) ZIF-8C. Reprinted from (CC BY license) [2].

Regarding platinum-based and advanced catalyst systems, ZIF-8-derived carbons have proven to be excellent supports for platinum-based catalysts, addressing the dual challenges of reducing Pt loading while enhancing activity and durability. Liao et al. developed hollow Fe–NC supports through stress-induced shrinkage of PDA-coated Fe-doped ZIF-8, followed by Pt deposition, achieving improved mass activity of 1.34 A mgPt^−1^—6.77 times higher than commercial Pt/C—with improved durability showing only 14.18% mass activity loss after 20,000 cycles compared to 76% loss for Pt/C [55]. DFT calculations revealed that strong Pt–FeNC interactions enhance Pt anchoring and weaken *OH adsorption on both Pt and Fe sites. Huang et al. employed a cooperative template strategy using CTAB and sodium laurate surfactants to create mesoporous carbon (p-ZIF-8-C) with pore sizes of 5–15 nm, achieving half-wave potential of 0.915 V and power density of 801 mW cm^−2^ in H_2_/air fuel cells, outperforming commercial Pt/C [56]. Hanif et al. developed PtNiCo/NC catalysts from NiCo-ZIF precursors, demonstrating 15% performance improvement over commercial Pt/C with peak power density of 1067 mW cm^−2^ at 70 °C and no degradation after 100 h of operation, attributed to synergistic effects of N-doped carbon and Pt/Pt alloy nanoparticles [57]. The transformation of ZIF-8 into huge-diameter carbon nanotubes (Fe-N-CNTs-H) by incorporating Fe-citrate enabled full utilization of active sites on both interior and exterior walls, achieving 665 mW cm^−2^ in H_2_-O_2_ PEMFC [58]. Park et al. demonstrated mesoporous nitrogen-doped carbon (Meso-ZIF-NC) with enhanced mesopore volume (0.513 cm^3^/g) and improved Pt nanoparticle dispersion, achieving half-wave potential of 0.82 V (Figure 4) [59]. Two-step pyrolysis of ZIF-8 functionalized with ammonium ferric citrate produced catalysts with ORR activities comparable to commercial 40 wt% Pt/C, achieving 391 mW cm^−2^ in PEMFC with only 12 mV shift after 10,000 cycles [60]. Xu et al. developed FeNC catalysts using ferrocene as an iron source via ball-milling and two-step pyrolysis, achieving onset potential of 0.95 V and maximum power density of 601 mW cm^−2^, with DFT revealing O species desorption as the rate-determining step [61].

Regarding durability and degradation mechanisms, the durability of ZIF-8-derived catalysts remains the primary barrier to commercial viability. Fe–N–C catalysts suffer rapid performance degradation (>80% loss within 35 h) primarily due to Fenton reactions generating reactive oxygen species that attack carbon supports and demetallate Fe–N_x_ active sites [45,52]. Stability enhancement strategies include: (i) Zr co-doping to suppress H_2_O_2_ formation [45,46]; (ii) graphitic carbon encapsulation to protect active sites [62]; (iii) CNT networks to enhance corrosion resistance [53]; and (iv) acid leaching to remove inactive metal species [54]. The translation from RDE to MEA performance remains challenging, as highlighted by Liu et al., who demonstrated that RDE results may not predict PEMFC performance, emphasizing the importance of actual fuel cell testing [42].

### 5.2. ZIF-8 for Direct Methanol Fuel Cells (DMFCs)

DMFCs offer advantages over hydrogen-fed PEMFCs, including liquid fuel handling, high energy density, and infrastructure compatibility. However, DMFC commercialization is hindered by two primary challenges: methanol crossover through the polymer electrolyte membrane, which poisons the cathode catalyst and reduces cell voltage, and the sluggish kinetics of methanol oxidation reaction (MOR) at the anode. ZIF-8 addresses both challenges through its molecular sieving properties (3.4 Å pore aperture effectively blocking methanol molecules at ~3.8 Å) and its ability to form catalytically active structures for MOR. This section critically analyzes ZIF-8-based materials for DMFC applications, separately addressing membrane applications and catalytic applications.

#### 5.2.1. Membrane Applications

ZIF-8 has emerged as an effective filler for polymer electrolyte membranes in DMFCs, leveraging its rigid microporous structure to suppress methanol crossover while enhancing proton conductivity through imidazole-mediated Grotthuss hopping. Guo et al. reported the first zwitter-MOF membrane by threading zwitterionic SBMA through ZIF-8 membranes via a self-confined growing method [66]. The sulfonate and quaternary ammonium groups on SBMA generated hydrogen-bond networks for ion transport pathways. The pSZ-11% membrane achieved proton conductivity of 3.62 × 10^−2^ S cm^−1^ at 75 °C and hydroxide conductivity of 2.52 × 10^−3^ S cm^−1^. Importantly, the well-intergrown ZIF-8 membrane provided efficient methanol blocking with permeability of 7.66 × 10^−9^ cm^2^ s^−1^ due to size sieving. DMFCs assembled with pSZ membranes showed good performance in both acidic and alkaline systems (Table 6).

Junoh et al. developed a novel porous PES/ZIF-8 composite electrolyte via dead-end filtration in situ growth, embedding ZIF-8 crystals within PES pore structures [67]. The composite achieved 85-fold enhancement in proton conductivity (17.9 × 10^−3^ S cm^−1^) compared to pristine PES. ZIF-8’s imidazole linkers acted as both proton donors and acceptors, facilitating Grotthuss mechanism proton transport through hydrophilic channels. The hydrophobic nature of ZIF-8 created tortuous pathways for methanol, reducing permeability to 3.88 × 10^−7^ cm^2^ s^−1^. Despite higher water uptake (164.61%), swelling ratio decreased 1.73-fold due to mechanical reinforcement from ZIF-8, demonstrating 6-fold and 2-fold higher selectivity than pristine PES and Nafion, respectively.

Liu et al. developed a functional nanofiber substrate by in situ growing ZIF-8 on PVDF nanofibers, followed by ionic liquid modification containing sulfonic acid groups [68]. The IL-ZIF-8@PVDF/SPEEK composite membrane exhibited proton conductivity of 161.92 mS cm^−1^ at 80 °C and peak power density of 114.86 mW cm^−2^ in DMFC, 1.28 times higher than Nafion 211. The IL modification improved compatibility between ZIF-8@PVDF and SPEEK, while the continuous PVDF nanofiber network established long-distance H^+^ transport pathways. The membrane demonstrated excellent methanol barrier performance (CCD: 58.18 mA cm^−2^ under 1 M methanol) and mechanical strength (39 MPa dry, 28 MPa wet). Durability testing over 320 h showed only 6.3% OCV attenuation. The synergistic effect of ZIF-8’s imidazole groups, IL’s -SO_3_H groups, and SPEEK’s sulfonic acid groups created a rich hydrogen bonding network for efficient proton transport via Grotthuss mechanism.

#### 5.2.2. Catalytic Applications

ZIF-8-based materials have demonstrated improved catalytic activity for MOR and ORR in DMFCs, functioning as both noble metal supports and metal-free catalysts (Table 7).

Hoseini et al. presented the first example of ZIF-8 nanostructured film synthesized at an oil–water interface at room temperature [69]. The self-assembly method produced a high-quality, uniform thin film without Nafion or binders. The ZIF-8 film exhibited excellent MOR activity with current density of 32.7 mA cm^−2^, higher than Pt film (30.6 mA cm^−2^). The long-term poisoning rate (δ = 0.1%) was comparable to expensive Pt-based catalysts. The film achieved power density of 15.4 mW cm^−2^ compared to MEA/PtRu (6.8 mW cm^−2^). Askarisarvestani et al. demonstrated a facile liquid–liquid interface method for synthesizing Pt@ZIF-8 thin films using three strategies: ship-in-a-bottle (SIB), bottle-around-the-ship (BAS), and in situ [70]. The in situ strategy produced Pt@ZIF-8 nanorods without templates, exhibiting the highest BET surface area (1643 m^2^/g) and electrocatalytic performance for methanol oxidation (48.10 mA cm^−2^). Bae et al. developed Pt-supported catalysts using ZIF-8 derived porous carbon (CZIF8) and nitrogen-doped MWCNT composites [71]. The Pt-NCNT@CZIF8 composite exhibited ECSA of 93.33 m^2^ g^−1^, significantly higher than Pt-CNT (55.23 m^2^ g^−1^). For methanol oxidation, the forward peak current density was 4.32 mA cm^−2^ with I_F/I_R ratio of 1.75, indicating improved CO tolerance.

**Figure 5:**
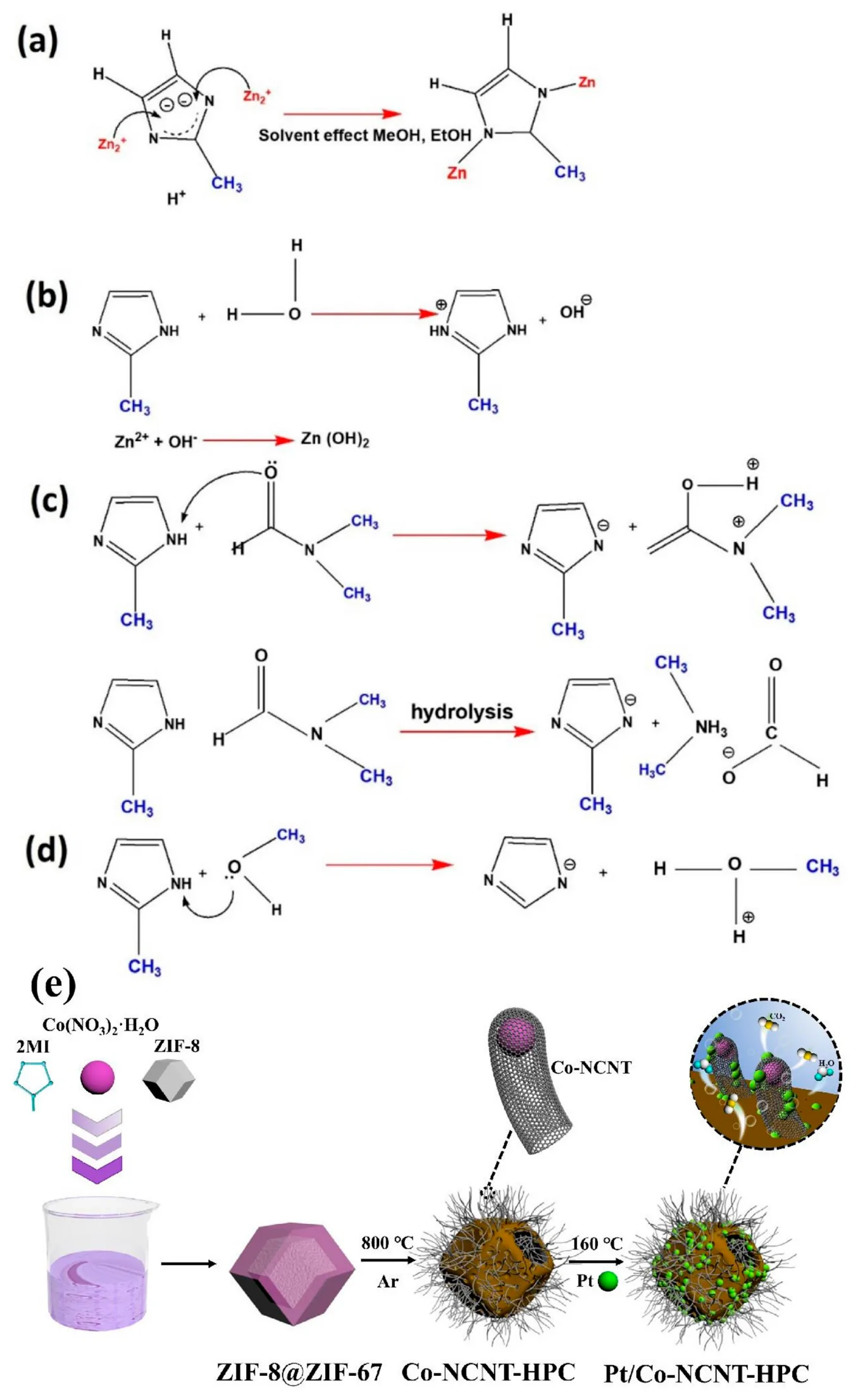
(**a**–**d**) Synthesis of ZIF-8 in methanol and ethanol (**a**), synthesis of ZIF-8 in water (**b**), HMIM deprotonated in DMF (**c**), and HMIM deprotonation in methanol (**d**). Reprinted from (CC BY license) [10]. (**e**) The preparation process for Pt/Co-NCNT-HPC catalysts is illustrated schematically. Reprinted from (CC BY license) [73].

Sui et al. developed a porous N-doped graphene aerogel (GA-CNx-ZIF-8) via hydrothermal self-assembly of ZIF-8 and graphene oxide [72]. The resulting Pt/GA-CNx-ZIF-8 catalyst achieved ECSA of 89.0 m^2^/g Pt, significantly higher than Pt/GA-900 (52.7 m^2^/g) and commercial Pt/C (48.7 m^2^/g). MOR activity was 1.8 times higher than commercial Pt/C, with enhanced durability (77.9% retention after 1000 cycles vs. 59.2% for Pt/C). Wang et al. developed Co embedded into nitrogen-doped carbon nanotube hollow porous carbon (Co-NCNT-HPC) derived from ZIF-8@ZIF-67 as a Pt catalyst support (Figure 5) [73]. The Pt/Co-NCNT-HPC800 catalyst exhibited improved mass activity (416.2 mA/mgPt) compared to commercial Pt/C (153.6 mA/mgPt), attributed to the synergistic effect of ZIF-8 (creating pores via Zn evaporation) and ZIF-67 (providing Co for CNT growth).

Chen et al. synthesized ZIF-8-derived nitrogen-enriched carbon frameworks on expanded graphite (Z8-NCF-P/EGs) as metal-free ORR catalysts for acidic DMFCs [74]. The PVP-derived carbon thin layer protected active sites from deterioration during pyrolysis. Z8-NCF-P/EG-10 exhibited ORR activity comparable to Pt/C (10 wt%) with Eonset = 0.663 V vs. SCE, JL = 6.3 mA cm^−2^, and E1/2 = 0.557 V in acidic electrolyte. The catalyst showed improved methanol tolerance and stability (only 14 mV negative shift after 5000 cycles) compared to Pt/C (28 mV). Pyridinic N and graphitic N provided highly active sites, and the micro-mesoporous structure (S_BET = 666 m^2^ g^−1^) with 3D bulk networks facilitated rapid oxygen and mass transfer kinetics.

### 5.3. ZIF-8 for Anion Exchange Membrane Fuel Cells (AEMFCs)

Anion exchange membrane fuel cells (AEMFCs) offer several advantages over proton exchange membrane fuel cells, including the use of non-precious metal catalysts due to faster oxygen reduction reaction kinetics in alkaline media, and reduced membrane cost. However, AEMFCs face challenges including low hydroxide conductivity, CO_2_ poisoning of anion-exchange membranes, and limited membrane stability under alkaline conditions. ZIF-8 has emerged as an important material for AEMFC applications, serving both as a membrane filler to enhance ion conductivity and alkaline stability, and as a precursor for high-performance ORR catalysts. This section critically analyzes ZIF-8-based materials for AEMFC applications, separately addressing membrane and catalytic functions.

#### 5.3.1. Membrane Applications

ZIF-8 has demonstrated significant potential as a filler for anion exchange membranes, leveraging its hydrophobic nature, molecular sieving properties (3.4 Å pore aperture), and imidazole functionality to enhance ion conductivity, methanol blocking, and alkaline stability (Table 8).

**Figure 6:**
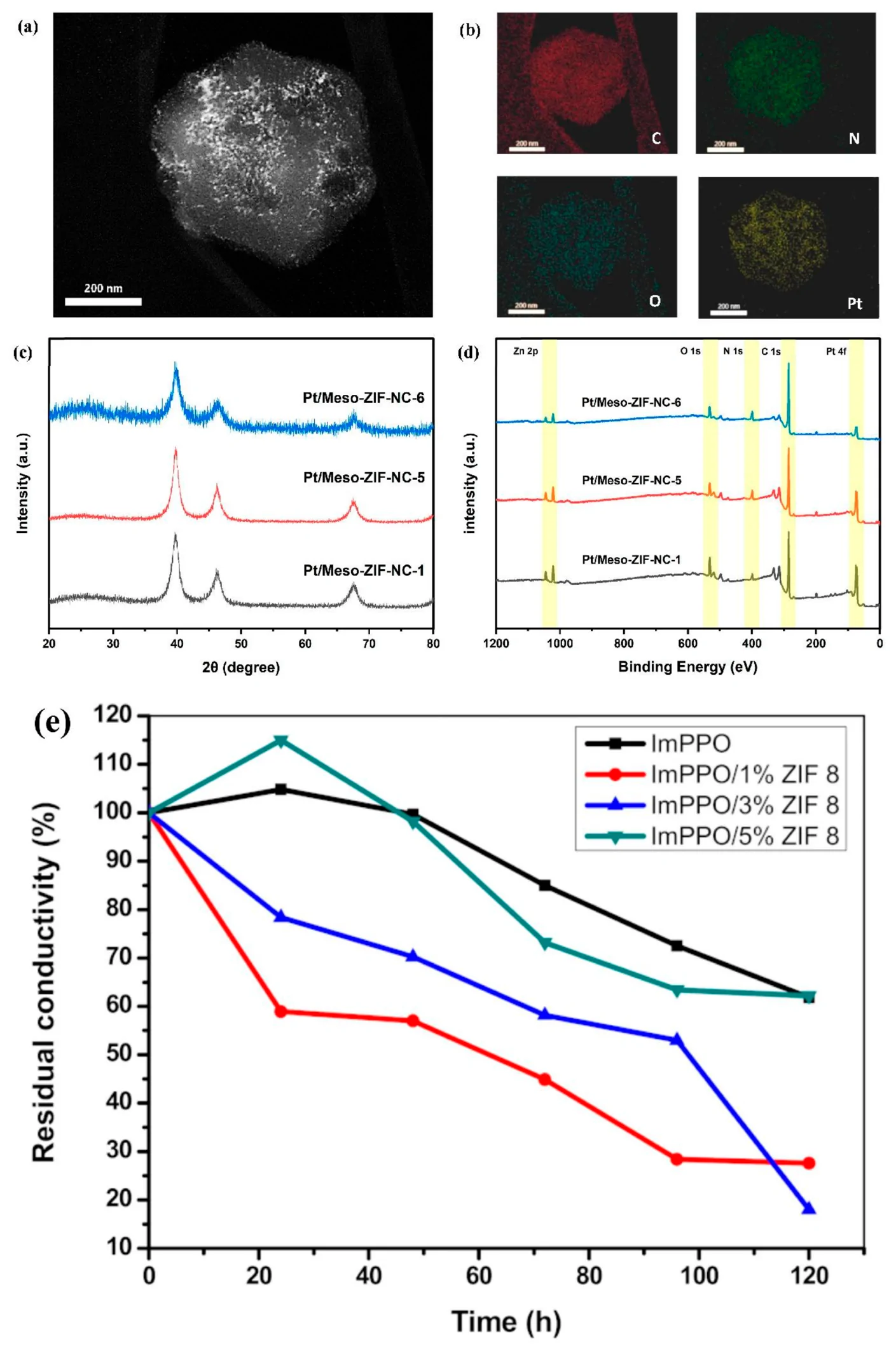
(**a**–**d**), STEM image (**a**) and elemental mapping images (**b**) of Pt/Meso-ZIF-NC-5, then XRD patterns (**c**) and XPS full spectrum (**d**) of Pt/Meso-ZIF-NC-1, Pt/Meso-ZIF-NC-5, and Pt/Meso-ZIF-NC-6. Reprinted from (CC BY license) [59]. (**e**) Durability of the IMPPO/ZIF-8 membrane toward alkaline exposure in 2 M aqueous NaOH. Reprinted from (CC BY license) [75].

Letsau et al. evaluated imidazolium-quaternized poly(2,6-dimethyl-1,4-phenylene oxide) (ImPPO) blended with ZIF-8 as anion exchange membranes [75]. The ImPPO/3%ZIF-8 membrane exhibited optimal properties: water uptake of 31%, swelling ratio of 15.1%, ion exchange capacity of 4.06 mmol/g, and ionic conductivity of 1.96 mS/cm. The hydrophobic ZIF-8 nanoparticles reduced excessive water uptake while enhancing alkaline stability (62% conductivity retention after 120 h) (Figure 6). The membrane achieved power density of 158.10 mW/cm^2^, surpassing commercial Fumasep FAS-PET-130 (109.49 mW/cm^2^). ZIF-8’s cavity size (3.4 Å) effectively blocks methanol crossover (methanol diameter 3.8 Å), demonstrating ZIF-8’s potential as a filler for improving AEM properties beyond traditional catalyst applications (Figure 6).

**Figure 7:**
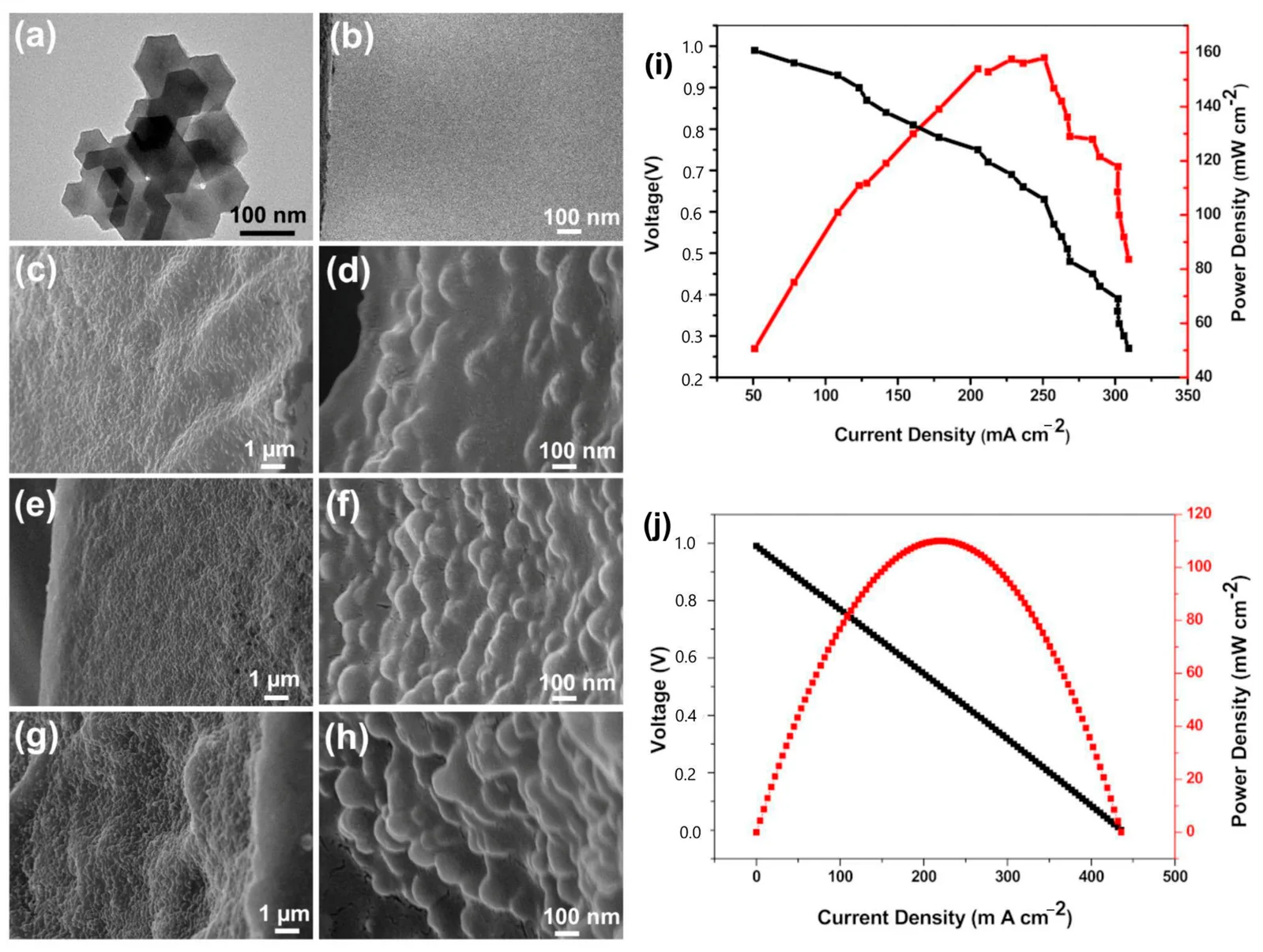
(**a**) Transmission electron microscopic image of pristine ZIF-8 nanoparticles; low and high magnification cross-sectional FESEM images of (**b**) PVA/GA, (**c**,**d**) PVA/25.4%ZIF-8/GA, (**e**,**f**) PVA/40.5%ZIF-8/GA, and (**g**,**h**) PVA/45.4%ZIF-8/GA composite membranes. Reprinted from (CC BY license) [76]. Polarization curves of (**i**) ImPPO/ZIF-8 and (**j**) commercial AEM using DMAFC at room temperature. Reprinted from (CC BY license) [75].

Hsu et al. developed swelling-resistant PVA/ZIF-8/GA composite membranes using water-based ZIF-8 synthesis and glutaraldehyde crosslinking [76]. The PVA/40.5%ZIF-8/GA achieved 190.5 mW/cm^2^ power density at 60 °C, 181% higher than the PVA/GA membrane. The GA crosslinking reduced volume swelling (<1.4%) and improved alkaline stability (0.19% conductivity loss after 168 h). ZIF-8 particles (3.4 Å pore size) selectively allow OH^−^ and water passage while blocking methanol (3.8 Å) (Figure 7). The pre-crosslinking approach before film casting preserved membrane integrity and conductivity, making these composites for direct alkaline methanol fuel cells with improved durability (Figure 7 and Table 8).

#### 5.3.2. Catalytic Applications

ZIF-8-derived catalysts have demonstrated improved ORR activity in alkaline media for AEMFC applications, often surpassing commercial Pt/C performance due to the favorable kinetics of ORR in alkaline environments and the high density of M–N_x_ active sites (Table 9).

Kumar et al. developed transition-metal-doped ZIF-8@CNT composite catalysts for AEMFCs [77]. The bimetallic Fe_1_Co_2–_ZNT-900 catalyst achieved improved ORR activity with half-wave potential of 0.847 V, surpassing commercial Pt/C (0.834 V). XPS analysis confirmed the presence of M–N_x_ active sites, with bimetallic catalysts containing the highest amount of pyridinic-N, pyrrolic-N, graphitic-N, and M–N_x_ species. In H_2_-O_2_ AEMFC testing, Fe_1_Co_2–_ZNT-900 delivered a maximum power density of 0.171 W cm^−2^ and current density of 0.326 A cm^−2^ at 0.5 V, approaching Pt/C performance (0.215 W cm^−2^). The hierarchical micro-mesoporous structure and FeCo nanoalloys contributed to the enhanced ORR kinetics.

Palm et al. introduced Fe, N, S tridoping via ball-milling and NH_3_ pyrolysis [78]. FeNSC2 exhibited E_1_/_2_ of 0.88 V, surpassing Pt/C (0.84 V), with low HO_2_^−^ yield and n close to 4. XPS confirmed successful S incorporation (0.1–0.5 at%) and high N content (6–7 at%) with pyridinic N (1.9–2.2 at%), Fe–N_x_ (0.3–0.6 at%), and graphitic N (0.6–0.8 at%). N_2_ physisorption revealed high surface area (1260 m^2^ g^−1^ for FeNSC2) with hierarchical micro-mesopores. Stability tests showed excellent durability with only 4 mV E_1_/_2_ shift after 10,000 cycles. The synergistic effect between N and S dopants enhanced ORR activity by creating larger asymmetrical spin and higher charge density. In AEMFC testing, FeNSC2 achieved P_max of 162 mW cm^−2^. The ball-milling approach offers a scalable synthesis method for producing high-performance ORR catalysts.

Freitas et al. demonstrated that pyrolysis temperature critically influences Zn removal from ZIF-8 and subsequent Fe–N_x_ active site formation [79]. The Fe–N–C-1000 catalyst exhibited improved ORR activity with E_1_/_2_ of 0.87 V, surpassing Pt/C performance in alkaline media. XPS analysis confirmed increased Fe–N coordination at higher temperatures, correlating with enhanced catalytic activity. The catalyst showed improved durability with 94% ECSA retention after 30,000 cycles, significantly outperforming Pt/C (35% retention). Methanol tolerance studies revealed improved resistance up to 2 M concentration, attributed to the Fe–N_x_ active sites’ selectivity. In AEMFC testing, Fe–N–C-1000 achieved competitive performance (149 mW cm^−2^) compared to Pt/C (148 mW cm^−2^). The material’s high methanol tolerance enabled improved ADMFC performance (49 mW cm^−2^) at 5 M methanol, demonstrating the practical viability of MOF-derived catalysts for next-generation alkaline fuel cells (Table 9).

### 5.4. ZIF-8 for Microbial Fuel Cells (MFCs)

MFCs represent a sustainable technology that converts chemical energy stored in organic matter directly into electrical energy through microbial metabolism, offering simultaneous wastewater treatment and energy recovery [41]. However, MFC commercialization is hindered by low power densities, high cathode costs due to platinum-based catalysts, and biofouling of electrodes. ZIF-8 and its derivatives have emerged as cathode catalysts for MFCs, leveraging their high surface area, abundant nitrogen functionality, and ability to form active M–N_x_ sites for efficient ORR under neutral pH conditions (Table 10) [41,80,81,82,83,84,85,86,87,88,89,90,91,92].

Unlike PEMFCs operating under acidic conditions (0.1–0.5 M H_2_SO_4_, pH ≈ 1) with strict temperature and humidity control, MFCs operate under mild, neutral-pH conditions (typically pH 6–8) at ambient temperatures (25–37 °C), with the cathode exposed to air and the anode immersed in wastewater containing complex organic substrates [80,86]. These fundamentally different operating conditions create distinct catalytic requirements: (i) the neutral pH environment favors different ORR mechanisms and active site configurations; (ii) the presence of microorganisms and organic matter introduces biofouling risks and mass transport limitations; (iii) the lower operating temperatures reduce kinetic rates, increasing the importance of high intrinsic activity; and (iv) the requirement for long-term stability (months rather than hours) places greater emphasis on durability in complex biological environments [82,84,91]. The primary performance metrics for MFC cathode catalysts are power density (mW/m^2^), which reflects actual electricity generation, COD (chemical oxygen demand) removal efficiency, which indicates wastewater treatment effectiveness, and long-term stability against biofouling and catalyst degradation [80,90]. These application-specific requirements guide the material design strategies discussed below.

This section systematically analyzes ZIF-8-based materials for MFC applications through six thematic areas: (1) material-design and synthesis strategies; (2) comparative analysis of Fe–N–C and related catalyst systems; (3) relationships between material properties and performance improvements; (4) relationships between ORR activity and MFC performance; (5) durability and biofouling; and (6) MFC-specific challenges and future directions. Table 10 provides a comprehensive summary of catalyst performance across the literature.

#### 5.4.1. Material Design and Synthesis Strategies

ZIF-8-based cathode catalysts for MFCs have been developed through five primary strategies, each offering distinct advantages for neutral-pH ORR and MFC-specific performance requirements.

Xue et al. first demonstrated that pristine ZIF-8, without pyrolysis or metal doping, can function as an efficient and sustainable ORR catalyst for MFCs [80]. This approach is unique among ZIF-8-based MFC catalysts, as most studies employ pyrolyzed derivatives. The as-synthesized ZIF-8 exhibited a hierarchical pore structure (micropores, mesopores, and macropores coexist), a large specific surface area (1416.19 m^2^/g), and a pore volume of 0.55 cm^3^/g. XPS analysis revealed abundant pyridinic nitrogen (81.03%) and N_x_–M bonds, which are crucial for the four-electron oxygen reduction pathway. The ZIF(0.5)-MFC achieved an improved maximum power density of 2103.46 mW/m^2^, 1.62 times higher than Pt/C-MFC, with stable operation exceeding 110 h. This study demonstrates that ZIF-8’s intrinsic nitrogen functionality and hierarchical porosity can enable efficient ORR without the need for high-temperature pyrolysis, offering a simpler and more energy-efficient synthesis route. However, the absence of transition metal active sites raises questions about the long-term stability of pristine ZIF-8 under prolonged MFC operation, where gradual framework degradation could occur.

The most extensively studied approach involves pyrolyzing Fe-doped ZIF-8 at temperatures between 700 and 1000 °C to generate atomically dispersed FeN_4_ active sites within nitrogen-doped carbon frameworks [82]. Wang et al. systematically investigated the effect of pyrolysis temperature (700, 800, 900 °C) on ORR activity and MFC performance [82]. The Fe–N–C-900 catalyst, pyrolyzed at 900 °C, exhibited the optimal balance between graphitization (enhanced electron conductivity) and active site preservation (FeN_4_ coordination), achieving an onset potential of 0.91 V and half-wave potential of 0.85 V in alkaline media, with a corresponding MFC power density of 1508 mW/m^2^. The critical insight from this work is the temperature-dependent trade-off: lower temperatures (700–800 °C) preserve more nitrogen functionality but yield lower graphitization and conductivity, while higher temperatures (≥1000 °C) can induce metal agglomeration and active site loss, despite improved conductivity.

To enhance ORR activity beyond Fe-only systems, researchers have explored bimetallic and co-doped catalysts. Kumar et al. investigated Fe and Co co-doped ZIF-8/MWCNT composites (FeCo–N–C@900) for neutral-pH ORR [86]. The bimetallic catalyst exhibited improved E_1_/_2_ of 0.71 V and J_lim of −5.62 mA/cm^2^ in 0.1 M PBS. Importantly, cyanide poisoning tests confirmed significant M–N_x_ site involvement in ORR, while XPS analysis revealed that bimetallic catalysts contain the highest amount of pyridinic-N, pyrrolic-N, graphitic-N, and M–N_x_ species compared to monometallic counterparts. Wang et al. demonstrated a Fe/Fe_3_C@NiNC catalyst derived from Fe, Ni co-doped ZIF-8 [83], where nickel doping optimized the electronic structure and increased pyridinic nitrogen content. The one-step pyrolysis approach generated carbon nanotubes that encapsulate Fe/Fe_3_C nanoparticles, providing a protective barrier that significantly improves long-term durability. The MFC assembled with this catalyst achieved a power density of 1600 mW/m^2^ and maintained stability for 120 h, outperforming Fe-only catalysts in durability tests.

To address mass transport limitations and enhance active site accessibility, several studies have incorporated conductive supports or created hybrid structures. Kumar et al. integrated ZIF-8-derived catalysts with MWCNTs, creating hierarchical micro-mesoporous structures that enhanced ORR kinetics by improving electron transport and facilitating oxygen diffusion to active sites [86]. Yang et al. developed TiO_2_-encased dual MOFs (ZIF-67/ZIF-8) via a two-step hydrothermal method [81], where the core–shell structure ensured structural integrity and high catalytic activity. TiO_2_@ZIF-67/ZIF-8 achieved P_max of 341.5 mW/m^2^, 1.30 times higher than ZIF-67/ZIF-8 (262.1 mW/m^2^) and 2.07 times higher than ZIF-67 (164.8 mW/m^2^). The synergy between dual MOFs (high stability, multiple active sites) and TiO_2_ (high surface area, catalytic activity) significantly improved ORR performance. Thulluru et al. synthesized the ZMG ternary composite (ZIF-8/MnO_2_/g-C_3_N_4_) without pyrolysis [91], achieving 2.13 times higher EASA than Pt/C with comparable charge transfer resistance (R_ct = 27.27 Ω vs. 27.42 Ω for Pt/C). Cost analysis revealed that ZMG is 48-fold cheaper than Pt/C, highlighting the economic viability of non-pyrolyzed composites for MFC applications where capital cost is a critical consideration.

To maximize active site exposure and prevent particle agglomeration, Guo et al. developed 1D carbon nanofibers with hollow beaded Co–N_x_ sites via electrospinning and pyrolysis [87]. PAN–Co–ZIF-8–C–A achieved P_max of 888.3 mW/m^2^ (1.19 times Pt/C). The 1D structure prevented particle agglomeration and enhanced active site exposure. Bpy poisoning tests confirmed that ORR activity originates from Co–N_x_ sites, and the catalyst achieved 89.8% COD removal, exceeding Pt/C (87.6%). Meng et al. developed interconnected Co–Zn/NC frameworks by in situ growth of ZIF-67 and ZIF-8 on nano-microfibrillar cellulose (MFC), followed by pyrolysis [92]. The Co–Zn/NC-30% catalyst exhibited excellent ORR activity (E_0_ = 0.974 V, E_1_/_2_ = 0.858 V), surpassing commercial Pt/C in alkaline media, with 90% current retention after 10,000 s.

#### 5.4.2. Comparative Analysis of Fe–N–C and Related Catalyst Systems

Fe–N–C catalysts represent the most extensively studied class of ZIF-8-derived MFC catalysts, with multiple studies examining variations in synthesis conditions, metal sources, and doping strategies. This subsection provides a systematic comparison to identify optimal design principles.

Shen et al. conducted a systematic investigation of how different Fe sources (Fe(acac)_3_, FePc, Fe(Gly)_2_) influence the ORR activity of Fe–NC catalysts derived from ZIF-8 [84]. The key finding is that Fe(acac)_3_ with a molecular size (9.7 Å) matching the ZIF-8 cavity (11.6 Å) enables complete encapsulation of the Fe precursor within the ZIF-8 framework, resulting in improved ORR activity exceeding commercial Pt/C. In contrast, FePc and Fe(Gly)_2_, with different molecular sizes, showed limited encapsulation and correspondingly lower activity. The Fe(A)–NC catalyst achieved 1917 mW/m^2^ in MFCs, significantly outperforming catalysts derived from other Fe sources. This study emphasizes the critical importance of precursor molecular size matching the MOF cavity, a design principle that may be transferable to other MOF-derived catalyst systems. Shen et al. further addressed environmental concerns by synthesizing ZIF-L in aqueous solution using Fe(acac)_3_ as the iron source [85]. Fe(A)–NC(L) showed improved ORR activity with E_1_/_2_ of 0.709 V (PBS) and 0.874 V (KOH), surpassing 20% Pt/C (0.687 V and 0.869 V). In MFC testing, Fe(A)–NC(L) achieved P_max of 1949 mW/m^2^, significantly higher than Pt/C (1612 mW/m^2^), with COD removal reaching 91.1%. This demonstrates that aqueous synthesis routes can achieve performance comparable to or better than conventional DMF-based methods while eliminating toxic solvents.

Wang et al. systematically varied pyrolysis temperature from 700 to 900 °C for Fe-doped ZIF-8 [82]. The Fe–N–C-900 catalyst, pyrolyzed at 900 °C, exhibited the best ORR activity (E_onset = 0.91 V, E_1_/_2_ = 0.85 V) and MFC performance (1508 mW/m^2^). This temperature-dependent behavior can be attributed to the following factors: (i) at 700–800 °C, nitrogen content is higher but graphitization is limited, resulting in lower conductivity; (ii) at 900 °C, optimal balance between nitrogen retention and graphitization is achieved; (iii) above 900 °C, Fe species aggregate into metallic nanoparticles, reducing the density of atomically dispersed FeN_4_ active sites. This trend is consistent with findings in the PEMFC catalyst literature, but MFCs may tolerate a broader temperature range due to the less demanding acidic environment.

Several studies have investigated bimetallic systems to enhance ORR activity and durability. Kumar et al. compared Fe-only, Co-only, and Fe–Co bimetallic catalysts derived from ZIF-8/MWCNT composites [86]. The Fe_1_Co_2_–ZNT-900 catalyst exhibited the highest ORR activity with E_1_/_2_ of 0.847 V, surpassing commercial Pt/C (0.834 V). XPS analysis confirmed that bimetallic catalysts contain the highest amount of pyridinic-N, pyrrolic-N, graphitic-N, and M–N_x_ species, suggesting that the presence of both Fe and Co promotes the formation of a greater density of active sites. In MFC testing, Fe_1_Co_2_–ZNT-900 delivered P_max of 1556.9 mW/m^2^, approaching Pt/C (1681.2 mW/m^2^), with 87.3% COD removal efficiency and 91.4% current retention after 10 h. The synergistic effect is attributed to (i) the creation of additional M–N_x_ sites from both metals, (ii) optimized electronic structure of the carbon matrix, and (iii) enhanced stability through the protective effect of graphitic carbon shells.

While Fe–N–C catalysts dominate the literature, copper-based ZIF-8 derivatives have also shown promise. Lai et al. synthesized Cu–N/C@Cu composites via a two-step pyrolysis strategy using Cu-ZIF-8 as a precursor [88]. The optimized Cu–N/C@Cu-2 catalyst achieved a half-wave potential of 0.67 V vs. RHE in neutral solution, close to commercial Pt/C (0.70 V). MFCs reached a maximum power density of 581 ± 13 mW/m^2^, surpassing Pt/C (499 ± 13 mW/m^2^). Interestingly, the copper nanoparticles effectively inhibited cathodic biofilm contamination, maintaining stable performance over 700 h. This antibacterial property is a unique advantage of copper-based catalysts for MFCs, where biofouling is a significant performance-limiting factor. Qi et al. fabricated CuCo–MOF@ZIF-8 composite catalysts using two-step hydrothermal and dual-solution methods [89]. The dual-solution method (CuCo–MOF@ZIF-8-2) achieved maximum power density of 246.38 mW/m^2^, 2.49 times higher than ZIF-8 (98.72 mW/m^2^), with stable voltage of 365 mV over 12 days. The Cu-based catalysts generally show lower absolute power densities than Fe-based systems, but their anti-biofouling properties may compensate for this deficit in long-term MFC operation.

#### 5.4.3. Relationships Between Material Properties and Performance

Understanding the structure–performance relationships in ZIF-8-derived MFC catalysts is essential for rational catalyst design. This subsection analyzes how specific material properties correlate with ORR activity and MFC power density.

High specific surface area is generally associated with higher ORR activity, as it provides a greater number of accessible active sites. Xue et al. reported a surface area of 1416.19 m^2^/g for pristine ZIF-8, which contributed to its improved MFC performance (2103.46 mW/m^2^) [80]. However, the relationship is not strictly linear: the Fe(A)–NC catalyst [84] exhibited high MFC performance (1917 mW/m^2^) despite a surface area of 962 m^2^/g, while some catalysts with higher surface areas showed lower performance due to poor active site distribution or limited mass transport. The hierarchical pore structure (coexistence of micropores, mesopores, and macropores) appears more critical than total surface area alone, as macropores facilitate mass transport to active sites located within micropores. This is particularly important in MFCs, where oxygen diffusion through the cathode and biofouling layers can be rate-limiting.

XPS analysis across multiple studies reveals a strong correlation between specific nitrogen configurations and ORR activity. Pyridinic nitrogen (N) and M–N_x_ species are consistently identified as the most active sites for the four-electron ORR pathway [80,82,86]. Xue et al. reported high pyridinic nitrogen content (81.03%) in pristine ZIF-8, which contributed to its high MFC performance without requiring metal doping [80]. In pyrolyzed catalysts, the relative proportions of pyridinic-N, pyrrolic-N, graphitic-N, and M–N_x_ depend on pyrolysis temperature and metal doping. Bimetallic Fe–Co catalysts show higher total N content (up to 9 at%) and higher M–N_x_ content than monometallic catalysts, correlating with enhanced ORR activity [86].

The degree of graphitization, which increases with pyrolysis temperature, affects electron conductivity and, consequently, ORR kinetics. However, higher graphitization is often accompanied by lower nitrogen content due to thermal decomposition of nitrogen functionalities. The optimal pyrolysis temperature (900 °C) represents a balance between these two competing factors [82]. Some studies have incorporated conductive supports such as MWCNTs to enhance conductivity without compromising nitrogen content [86]. This strategy has proven effective in improving MFC performance by reducing internal resistance and facilitating electron transfer from active sites to the external circuit.

While few studies have directly quantified M–N_x_ active site density in MFC catalysts, indirect evidence from cyanide poisoning tests suggests that higher M–N_x_ density correlates with better ORR performance [86]. The use of Fe(acac)_3_ as an iron source with molecular size matching the ZIF-8 cavity enables complete encapsulation, which likely leads to higher Fe loading and, consequently, higher FeN_4_ active site density after pyrolysis [84]. This design principle—ensuring uniform distribution of metal precursors within the MOF framework—appears critical for maximizing active site density.

#### 5.4.4. Relationships Between ORR Performance and MFC Performance

A critical question for MFC catalyst development is the extent to which ORR activity measured in half-cell configurations (RDE/RRDE) predicts MFC performance in full-cell configurations. Analysis of the literature reveals both correlations and discrepancies that deserve careful consideration.

In general, catalysts with higher ORR half-wave potentials (E_1_/_2_) and higher kinetic currents (J_k) tend to exhibit higher MFC power densities. For example, the Fe_1_Co_2_–ZNT-900 catalyst with E_1_/_2_ of 0.847 V achieved P_max of 1556.9 mW/m^2^ [86], while the ZIF-8-derived carbon catalysts with E_1_/_2_ of 0.86 V achieved 2535 mW/m^2^ [90], representing the highest reported MFC performance. However, the correlation is not perfect; some catalysts with comparable ORR activity show significant differences in MFC performance due to factors that are not captured by RDE measurements:

(i) RDE measurements are conducted in well-mixed liquid electrolytes, where oxygen diffusion is relatively rapid. In MFC cathodes, oxygen must diffuse through the gas diffusion layer, the catalyst layer, and possibly a biofilm layer, creating mass transport limitations that are not reflected in RDE data. Catalysts with hierarchical porosity (macropores for oxygen transport, mesopores for ion transport, micropores for active site accessibility) tend to outperform microporous catalysts in MFCs, even if their intrinsic ORR activity is similar [80,84,90].

(ii) The presence of microorganisms and organic matter in MFC cathodes can lead to biofilm formation on the catalyst surface, blocking active sites and increasing mass transport resistance. ORR measurements in clean electrolytes do not capture this effect. Copper-based catalysts, which exhibit antibacterial properties, show better performance retention in long-term MFC operation than their ORR activity would suggest [88].

(iii) MFC performance depends on the catalyst loading (mg/cm^2^), the type of current collector, and the membrane-electrode assembly configuration. Variations in these factors across studies complicate direct comparisons, even when similar ORR catalysts are used.

(iv) MFCs operate with actual wastewater (or synthetic substrates) at various pH values, ionic strengths, and temperatures. These conditions can differ substantially from the controlled conditions in RDE measurements (0.1 M PBS, room temperature, clean electrolyte), leading to discrepancies in catalyst performance.

These observations underscore the importance of full-cell MFC testing for evaluating catalyst performance, despite the convenience and reproducibility of RDE measurements. The most catalysts should be tested under conditions that closely mimic real-world MFC operation to assess their practical viability.

#### 5.4.5. Durability and Biofouling

Long-term stability is a critical requirement for MFC cathodes, as MFCs are designed for continuous operation over months or even years. Durability data from the literature are presented in Table 10 and summarized below.

The highest reported durability was achieved by Yang et al. with the Zn/DAFP-800 catalyst, which maintained 93.62% of initial performance after 30 days of operation [90]. This improved stability was attributed to the hollow structure of the catalyst, which enhances mass transport and reduces fouling, as well as the multi-heteroatom doping that stabilizes active sites against degradation. Lai et al. reported stable operation over 700 h (approximately 29 days) for the Cu–N/C@Cu-2 catalyst [88], which maintained performance due to the antibacterial properties of copper nanoparticles that inhibited cathodic biofilm formation. Wang et al. demonstrated 120 h of stability for the Fe/Fe_3_C@NiNC catalyst [83], where the encapsulation of Fe/Fe_3_C nanoparticles within carbon nanotubes provided a protective barrier against degradation.

Several studies reported shorter durability tests (8–12 days) [81,89], with stable voltage output maintained for the duration of testing. However, extended durability data beyond 30 days are scarce, representing a significant gap in the literature. For MFCs to be commercially viable, catalysts must demonstrate stable performance for at least 3–6 months under actual wastewater conditions.

Biofouling of cathodes is a major challenge in MFCs, as microbial attachment and biofilm formation increase mass transport resistance and can poison active sites. Two main strategies have been employed to address biofouling in ZIF-8-derived catalysts:

(i) Lai et al. demonstrated that copper nanoparticles incorporated into Cu–N/C@Cu catalysts effectively inhibit cathodic biofilm contamination, maintaining stable performance over 700 h [88]. The antibacterial properties of copper nanoparticles are well-established, and their incorporation into ZIF-8-derived catalysts provides a dual function: ORR catalysis and biofilm prevention.

(ii) Yang et al. developed hollow Zn/multi-heteroatom co-doped carbon catalysts that showed excellent performance retention over 30 days [90]. The hollow structure reduces the surface area available for microbial attachment, while the hydrophobic surface chemistry discourages biofilm formation.

Despite these strategies, systematic studies of biofouling mechanisms and their mitigation remain limited. Future research should include: (i) characterization of biofilm composition on ZIF-8-derived catalysts, (ii) investigation of surface chemistry modifications that discourage microbial attachment, and (iii) development of protocols for catalyst regeneration in MFCs.

#### 5.4.6. MFC Specific Challenges and Future Directions

Based on the analysis above, several MFC-specific challenges and future research directions can be identified:

While many ZIF-8-derived catalysts show excellent initial performance, long-term stability data (>6 months) are lacking. Future studies should prioritize extended durability testing under actual wastewater conditions, including variable temperature, pH, and organic loading.

The discrepancies between ORR activity measured in RDE and MFC power density highlight the need for more comprehensive characterization, including (i) ex situ electrochemical testing under conditions that mimic MFC catholyte (neutral pH, presence of microorganisms), (ii) in situ electrochemical measurements during MFC operation, and (iii) development of standardized MFC testing protocols to enable fair catalyst comparison.

The 48-fold cost advantage of ZMG composite over Pt/C demonstrates the economic potential of ZIF-8-based catalysts [91]. However, the synthesis costs of many ZIF-8-derived catalysts remain relatively high due to the use of organic solvents (DMF), high-temperature pyrolysis, and expensive precursors. Future work should focus on: (i) aqueous synthesis routes that eliminate toxic solvents [85], (ii) low-temperature synthesis or pyrolysis-free approaches [80], (iii) utilization of biomass-derived precursors, and (iv) continuous-flow synthesis for large-scale production.

The degradation of ZIF-8-derived catalysts in MFCs involves multiple mechanisms: (i) carbon corrosion, (ii) active site demetallation, (iii) loss of nitrogen functionality, (iv) biofouling, and (v) mechanical degradation of the catalyst layer. Systematic studies using ex situ and operando characterization techniques (XPS, TEM, Raman spectroscopy, electrochemical impedance spectroscopy) are needed to identify the dominant degradation pathways under MFC operating conditions and guide the design of more durable catalysts.

The integration of ZIF-8-derived catalysts into MFC cathode assemblies requires optimization of: (i) catalyst loading (typically 0.5–2 mg/cm^2^), (ii) binder composition and ratio (Nafion or PTFE), (iii) gas diffusion layer properties, and (iv) membrane–electrode interface. Systematic studies of these factors, similar to those conducted for PEMFC MEAs, would significantly advance MFC performance.

While Fe–N–C catalysts dominate the literature, other materials with potential advantages for MFCs should be explored, including: (i) earth-abundant non-Fe catalysts (e.g., Mn, Cu) with better stability and lower toxicity, (ii) metal-free heteroatom-doped carbons with high nitrogen content, (iii) bifunctional ORR/OER catalysts for MFCs with in situ oxygen generation, and (iv) catalysts with intrinsic anti-biofouling properties.

### 5.5. ZIF-8 for Zinc–Air Batteries

Zinc–air batteries (ZABs) have attracted significant attention as next-generation energy storage devices due to their high theoretical energy density (1086 Wh kg^−1^), low cost, environmental friendliness, and inherent safety. However, the practical application of ZABs is hindered by the sluggish kinetics of the oxygen reduction reaction (ORR) during the discharge and oxygen evolution reaction (OER) during charge, necessitating efficient bifunctional catalysts. ZIF-8-derived materials have emerged as cathode catalysts for ZABs, leveraging their high surface area, nitrogen-doped carbon frameworks, and transition metal active sites to achieve excellent ORR activity and durability. This section critically analyzes ZIF-8-based materials for zinc–air battery applications, focusing on catalyst design strategies and battery performance metrics (Table 11).

Wang et al. presented Fe-Fe_3_C embedded Fe–N–C catalysts derived from dual MOFs (MIL-100(Fe) and ZIF-8) for ORR applications in PEMFC and Li-O_2_ batteries [93]. The optimal catalyst achieved half-wave potentials of 0.88 V (alkaline) and 0.79 V (acidic), with peak power density of 0.76 W cm^−2^ in PEMFC. DFT calculations revealed that Fe_3_C enhances oxygen affinity while metallic Fe promotes *OH desorption. The catalyst outperformed Pt/C in durability and methanol tolerance, demonstrating the importance of Fe-Fe_3_C@Fe–N–C dual active sites.

Liu et al. synthesized Fe,Ni-co-doped ZIF-8 derived in situ grown carbon nanotubes (Fe–Ni/CN) as bifunctional catalysts for zinc–air battery-based self-powered electrochemical glucose sensors [94]. Fe–Ni/CN exhibited improved ORR performance with half-wave potentials of 0.892 V (alkaline) and 0.810 V (neutral), outperforming 20 wt% Pt/C. The neutral ZAB assembled with Fe–Ni/CN showed power density of 63.6 mW cm^−2^, specific capacity of 700 mAh g^−1^, and excellent durability (>120 h cycling). The FeNi alloy nanoparticles induced carbon nanotube growth, creating extensive electron transfer pathways. XPS revealed graphitic-N and pyridinic-N as predominant species. The ZAB-type SPES achieved a glucose detection range of 0.01–200 μM with LOD of 3.34 nM, with recoveries of 95.4–104.7% in human sweat samples, demonstrating the versatility of ZIF-8 derivatives beyond traditional fuel cell applications.

Yan et al. presented a facile NaCl template strategy for synthesizing nitrogen-doped hollow carbon polyhedrons (NHCP) from ZIF-8 [95]. The NaCl acts as a confining template preventing nitrogen loss, an intercalating agent creating mesopores, and a promoter of graphitization. NHCP-1000 exhibited improved ORR activity (E_1_/_2_ = 0.86 V) and Zn–air battery performance (272 mW cm^−2^, 160 h stability). The hollow porous structure with high surface area (1240.8 m^2^/g) and high graphitic/pyridinic N content enabled improved electrocatalytic performance.

Xuan et al. demonstrated a NaCl-assisted exfoliation strategy to synthesize 3D nitrogen-doped hierarchical porous carbons (NHPCs) from ZIF-8 polyhedrons [96]. The molten NaCl serves dual functions as a template wrapping carbon and as an intercalating agent penetrating through carbon via capillarity, promoting nanosheet formation and preventing aggregation. The optimal NHPC1.3-900 exhibited excellent ORR performance with E_1_/_2_ of 0.87 V, better than Pt/C, and better Zn–air battery performance (207 mW cm^−2^). The hierarchical porous structure (micro-, meso-, and macropores) with high surface area (1302 m^2^/g) and suitable nitrogen doping (pyridinic and graphitic N) enabled fast mass transfer and exposed active sites.

### 5.6. ZIF-8 for Supercapacitors

Supercapacitors have emerged as energy storage devices bridging the gap between conventional capacitors and batteries, offering high power density, rapid charge–discharge capability, and improved cycle stability. The electrochemical performance of supercapacitors depends critically on the electrode materials’ surface area, porosity, electrical conductivity, and electrochemical stability. ZIF-8 and its derivatives have attracted significant interest as electrode materials for supercapacitors, leveraging their high surface area, tunable porosity, and ability to form conductive carbon frameworks upon pyrolysis. This section critically analyzes ZIF-8-based materials for supercapacitor applications, focusing on specific capacitance, rate capability, and cycling stability (Table 12).

Sharma and Chand synthesized pristine ZIF-8 via a simple, acid-free solvothermal route at 150 °C for 4 h, producing 52 nm cubic-phase nanocrystals with rectangular plate morphology [97]. Electrochemical characterization in 6 M KOH revealed pseudocapacitive behavior with a b-value of 0.74 from CV analysis, indicating diffusion-controlled charge storage. The material achieved specific capacitance of 35.38 F/g at 1 A/g and 42.22 F/g at 10 mV/s. EIS analysis showed charge transfer resistance of 254 Ω and series resistance of 1.73 Ω. This study demonstrated that pure ZIF-8, without carbonization or composite formation, can function as an effective supercapacitor electrode material, leveraging its high porosity (surface area ~1150 m^2^/g) and intrinsic electrochemical activity.

Karim et al. presented a novel bio-inspired approach combining ZIF-8-derived ZnO/C with electrochemically active biofilm (EAB)-mediated Ag nanoparticle synthesis [98]. The EAB method created Ag@ZnO/C with enhanced surface area (68 m^2^/g vs. 13.5 m^2^/g) and excellent supercapacitor performance (921 F g^−1^ at 1 A g^−1^). The acidic environment during biofilm synthesis decreased particle size and increased mesoporosity. The material demonstrated improved cycling stability with 95% capacitance retention after 9000 cycles. This study demonstrated the synergy between MOF-derived materials and biological synthesis for energy storage applications, providing an environmentally friendly approach for producing high-performance supercapacitor electrodes.

### 5.7. ZIF-8 for Water Splitting (HER/OER)

Electrochemical water splitting represents a sustainable approach for hydrogen production, comprising two half-reactions: the hydrogen evolution reaction (HER) at the cathode and the oxygen evolution reaction (OER) at the anode. However, the practical application of water splitting is hindered by the sluggish kinetics of both reactions, necessitating efficient and durable electrocatalysts. ZIF-8-derived materials have emerged as electrocatalysts for water splitting, leveraging their nitrogen-doped carbon frameworks, high surface area, and ability to form active metal-nitrogen sites and conductive carbon structures. This section critically analyzes ZIF-8-based materials for water splitting applications, covering both OER and HER catalysts (Table 13).

Sreekanth et al. synthesized ZIF-8@CoFe_2_O_4_ composites with varying CoFe_2_O_4_ concentrations (ZCFO-1, ZCFO-2, ZCFO-3) for both methanol oxidation and oxygen evolution reactions [99]. The ZCFO-1 composite (10 mg CoFe_2_O_4_) exhibited improved performance with higher current density for MOR and lower overpotential (330 mV) for OER with a Tafel slope of 84 mV dec^−1^. The enhanced activity stems from the synergistic effect between ZIF-8’s porous structure and CoFe_2_O_4_’s catalytic properties, along with increased electrochemically active surface area (55.29 mF g^−1^). Chronoamperometry tests confirmed excellent stability for over 6 h. This bifunctional capability makes ZIF-8@CoFe_2_O_4_ a non-precious metal alternative for both fuel cell anodes and water splitting applications.

Vattikuti et al. demonstrated the synthesis of C,N-ZIF/TiFe nanostructures by embedding carbon and nitrogen from ZIF-8 into TiO_2_/Fe_2_O_3_ semiconductors for overall water splitting [100]. The C,N-ZIF/TiFe catalyst exhibited improved OER activity with an overpotential of 290 mV (near RuO_2_ at 270 mV) and HER activity with 291 mV overpotential, achieving a low overall water splitting cell potential of 1.64 V at 10 mA cm^−2^. The thermal decomposition of ZIF-8 created onion-ring-like carbon structures that enhanced electrical conductivity and provided additional active sites. The C–N–Ti–O and N–Ti–O bonds improved charge transfer and stability. The catalyst demonstrated excellent durability over 14 h with maintained morphological features. This noble-metal-free electrocatalyst shows improved bifunctional performance, combining OER and HER activities for practical water splitting applications.

Huang et al. reported encapsulation of high-dispersed Rh nanoparticles in N-doped porous carbon polyhedrons (NPCP) derived from ZIF-8 [101]. Rh@NPCP exhibited pH-universal HER activity (η_10_ = 20 mV in alkaline, 23 mV acidic, 45 mV neutral) and excellent ORR performance (E_1_/_2_ = 0.84 V). The encapsulation strategy prevented Rh aggregation and detachment, ensuring improved stability compared to commercial Pt/C. This demonstrates ZIF-8-derived carbons as supports for noble metal catalysts.

### 5.8. ZIF-8 for Other Electrochemical Applications

Beyond the major application areas discussed in previous sections, ZIF-8 and its derivatives have demonstrated improved versatility across a diverse range of emerging electrochemical technologies, including glucose fuel cells, hydrogen peroxide fuel cells, photo-bioelectrocatalytic fuel cells, CO_2_ electroreduction, electrochemical sensors, hydrogen storage, and smart membranes. This section critically analyzes these emerging applications, highlighting the unique advantages of ZIF-8-based materials and identifying future research directions (Table 14).

#### 5.8.1. Hydrogen Peroxide Fuel Cells and Electrochemical Sensors

Ravipati and Badhulika developed a ZIF-8-infused PVA hydrogel with peony flower-like morphology via coprecipitation and freeze-drying for H_2_O_2_ fuel cells and electrochemical detection [102]. The hydrogel exhibited a high current density of 1.4 A/g with 89% capacity retention, demonstrating excellent electrocatalytic performance. As a sensor, it achieved a linear detection range of 0.01–10 mM with a LOD of 0.02 mM and sensitivity of 8.6 mA mM^−1^ cm^−2^. The porous hydrogel structure enhanced ion transport and active site accessibility, while the flexibility provided adaptability for various applications. The sensor showed excellent selectivity against common interferents (Cl^−^, Mg^2+^, Ca^2+^, urea, NO_3_^−^, SO_4_^2−^) and successful real-time analysis in tap water with ~100% recovery, offering a bifunctional platform for energy conversion and environmental monitoring.

#### 5.8.2. CO_2_ Electroreduction

Ye et al. demonstrated surface functionalization of ZIF-8 with ammonium ferric citrate to produce isolated Fe–N sites on the carbon matrix surface after pyrolysis [103]. The surface-functionalized catalyst (C-AFC@ZIF-8) showed significantly higher ORR activity than bulk-functionalized catalysts due to better active site exposure. The catalyst demonstrated high mass activity in acidic medium and improved selectivity toward CO_2_ electroreduction (93% CO Faradaic efficiency at −0.43 V). XANES and EXAFS confirmed isolated Fe-N_4_ coordination similar to FePc, with no Fe–Fe bonds, establishing ZIF-8-derived materials as an important candidate for CO_2_ conversion applications.

#### 5.8.3. Photo-Bioelectrocatalytic Fuel Cells

Qing et al. combined ZIF-8, carbon dots, and bilirubin oxidase on microalgal cells for photo-bioelectrocatalytic fuel cells [104]. ZIF-8/CDs/bilirubin oxidase@algae achieved current density of 1767 μA cm^−2^, 2.26-fold higher than CDs/bilirubin oxidase/algae. DFT calculations revealed O_2_ binding energy of −0.51 eV with charge transfer from imidazole to O_2_, confirming efficient O_2_ enrichment. The ZIF-8 coating enhanced oxygen diffusion (D = 3.98 × 10^−5^ cm^2^ s^−1^ vs. 2.04 × 10^−5^ cm^2^ s^−1^) and protected algae (86.8% viability vs. 69% for unprotected). The biocathode maintained 82.4% current density after 60 h. When coupled with Mo:BiVO_4_ photoanodes, the PBFC achieved P_max of 131.8 μW cm^−2^ with tetracycline as fuel and 74% degradation efficiency in 3 h, enabling energy recovery from wastewater treatment without mechanical aeration.

#### 5.8.4. Glucose Fuel Cells

Xu et al. utilized ZIF-8-derived nitrogen-doped porous carbon (NCZIF-8) as a catalyst support for Pt nanoparticles in glucose fuel cells [105]. The NCZIF-8 support (944 m^2^/g, 3D porous structure) promoted uniform dispersion of Pt NPs (2.7 nm) and prevented aggregation, enhancing durability (only 7.83% decay after 300 cycles). The catalyst exhibited comparative activity with pure Pt NPs despite only 10% Pt loading. Importantly, the porous architecture provided oxygen tolerance by depleting dissolved oxygen before it reached Pt active sites. The dual-chamber glucose fuel cell achieved P_max of 0.54 μW/cm^2^ with Voc of 323.8 mV, extending ZIF-8 applications to biofuel cells.

#### 5.8.5. Ethanol Oxidation

Gamal et al. developed a ZIF-8-derived nitrogen-doped carbon platform on rGO supporting α-Ni(OH)_2_ nanoparticles for ethanol oxidation [106]. The α-Ni(OH)_2_/ZNC@rGO catalyst exhibited excellent EOR activity with onset potential of 0.34 V and current density of 8.3 mA cm^−2^, outperforming commercial Pt/C. The α-phase Ni(OH)_2_’s disordered structure and large interlayer spacing facilitated ion–solvent intercalation, while rGO and N-doped carbon provided high conductivity. The catalyst maintained 92% stability after 900 cycles, demonstrating the potential of MOF-derived carbon-supported Ni-based catalysts as cost-effective alternatives for direct ethanol fuel cells.

#### 5.8.6. Smart Proton-Conductive Membranes

Li et al. presented a novel light-switchable proton conductive membrane by incorporating Ag nanoparticles and ssDNA into ZIF-8 [107]. The Ag-DNA@ZIF-8 membrane achieved an improved Off/On ratio of 6.6 × 10^5^, decreasing proton conductivity by five orders of magnitude under light illumination. The mechanism relied on localized surface plasmon resonance heating from Ag-NPs, which disrupted hydrogen-bonded networks between DNA and water molecules. Reversibility occurred when light was removed, allowing water molecules to rebind with DNA. This photoswitchable proton conduction surpassed previous systems by orders of magnitude in the switching factor.

Guo et al. demonstrated the first MOF-based proton conductive membrane for DMFC applications [108]. DNA molecules were threaded through ZIF-8 via solid-confined conversion, creating hydrogen-bond networks between DNA’s amidogen/phosphate groups and water molecules within ZIF-8 cavities. The DNA@ZIF-8 membrane achieved proton conductivity of 0.17 S cm^−1^ at 75 °C and 97% RH, the highest reported for MOF-based proton conductors. The small pore aperture (0.34 nm) effectively blocked methanol crossover (1.25 × 10^−8^ cm^2^ s^−1^), providing improved selectivity compared to Nafion. The membrane’s power density of 9.87 mW cm^−2^ in DMFC demonstrated practical viability.

#### 5.8.7. Hydrogen Storage

Wu and Zheng combined DFT calculations and experiments to evaluate GO incorporation on ZIF-8 for onboard hydrogen storage applications [109]. DFT calculations revealed adsorption energies of −0.1405 eV for 1GO@ZIF-8, significantly higher than pristine ZIF-8 (−0.0557 eV). The GO-2@ZIF-8 composite (2 wt% GO) exhibited optimal hydrogen storage performance with 3.44 wt% at 77.15 K and 5 MPa, 11.48% higher than pristine ZIF-8. Cycling tests showed minimal deterioration (0.033% variation after five cycles), and hydro-stability tests demonstrated only 0.32% surface area decrease after 5-day exposure to 76% RH at 21 °C, confirming enhanced stability for marine onboard applications.

#### 5.8.8. Metal-Free and Composite ORR Catalysts

Several studies have explored ZIF-8-derived metal-free and composite catalysts for ORR applications. Zhang et al. reported highly graphitized nitrogen-doped porous carbon nanopolyhedra (NGPCs) derived directly from ZIF-8 nanocrystals as metal-free ORR catalysts [110]. The optimized NGPC-1000-10 exhibited ORR activity with E_onset = −0.017 V vs. Ag/AgCl, J_L = 4.69 mA cm^−2^, n = 3.81, and improved methanol tolerance. Graphitic-N played a more important role in ORR than pyridinic-N.

He et al. developed atomically dispersed CoN_4_ catalysts with core–shell structure via surfactant-assisted MOF approach using F127 block copolymer [111]. Co–N–C@F127 exhibited ORR activity with E_1_/_2_ = 0.84 V vs. RHE, H_2_O_2_ yield < 2%, and power density of 0.87 W cm^−2^ in H_2_-O_2_ fuel cells. DFT calculations revealed that CoN_2+2_ sites (edge sites) are thermodynamically favorable for 4e^−^ ORR, while CoN_4_ sites (embedded) favor a 2e^−^ pathway. This represents the highest reported PGM-free and Fe-free catalyst performance.

Zou et al. presented a KCl-assisted pyrolysis method to prepare N-doped porous carbon nanosheets (NCNS) from ZIF-8 [112]. The optimal NCNS-10-900 exhibited a half-wave potential of 0.88 V, better than Pt/C (0.86 V), with only 17 mV degradation after 10,000 cycles. The high specific surface area (2495 m^2^/g) and hierarchical micro-/mesoporous structure exposed abundant active sites.

Bakhtavar et al. synthesized a ternary composite Co@NC/GCN-CNT by combining ZIF-8@ZIF-67 derived carbon, graphitic carbon nitride (GCN), and carbon nanotubes [113]. The optimized catalyst exhibited onset potential of −0.07 V vs. Ag/AgCl and current density of −8.5 mA cm^−2^, approaching commercial Pt/C performance with n = 3.96.

Zafar et al. developed bioinspired nitrogen-rich carbon coated ZIF-8 (NRC@ZIF-8) using nitrodopamine as the N source [114]. The catalyst exhibited improved half-wave potential of 0.91 V and 91.88% retention against methanol poisoning, surpassing commercial Pt/C. The NRC coating provided positively charged N atoms for enhanced O_2_ adsorption and promoted heterolytic O-O bond cleavage.

#### 5.8.9. Biomass-Derived and Eco-Friendly Catalysts

Li et al. presented an eco-friendly strategy using ZIF-8 as an activator for legume root nodule-derived carbon materials [115]. The in situ growth of ZIF-8 on pre-carbonized root nodules, followed by pyrolysis, yielded porous carbon with an improved surface area of 1459.27 m^2^ g^−1^. The catalyst RW@Z8(5) demonstrated ORR activity with half-wave potential of 0.720 V in acidic media, only 88 mV lower than Pt/C. The self-doping of heteroatoms (Fe, Mo, N, S) from the biomass contributed to catalytic activity, replacing harsh chemical activators like KOH and ZnCl_2_.

## 6. Critical Analysis and Future Perspectives

The literature screening process for this review revealed several recurring methodological weaknesses in studies on ZIF-8-based electrochemical materials. Addressing these issues would significantly enhance the reproducibility, comparability, and scientific rigor of future research.

Many studies omit critical synthesis parameters (e.g., precursor ratios, reaction time, heating/cooling rates) or characterization conditions (e.g., scan rates in CV, frequency ranges in EIS). We recommend following established reporting guidelines, including detailed synthesis protocols, complete characterization parameters, and raw data deposition where possible.

A common weakness is comparing novel catalysts to Pt/C under conditions that favor the non-precious catalyst (e.g., testing in alkaline media when Pt/C is optimized for acidic conditions) or using different loading levels that bias performance comparisons. Benchmarks should be tested under identical conditions, with catalyst loadings normalized to geometric or mass-specific basis, and comparisons should explicitly state the reference material’s commercial source and batch.

For fuel cell studies, many reports fail to specify critical MEA testing conditions: temperature, backpressure, relative humidity, gas flow rates, catalyst loading on both electrodes, and membrane pretreatment history. Without this information, cell performance cannot be reproduced or meaningfully compared. We recommend adopting standardized reporting protocols.

Many studies report initial performance only or use durability tests that are too short (<100 cycles or <10 h) to provide meaningful insights. We recommend accelerated stress tests (30,000 cycles for ORR catalysts) and extended chronoamperometry (>24 h) to capture degradation trends, with performance metrics reported at multiple time points.

Many studies overstate catalyst performance by reporting RDE results as predictive of MEA performance, despite well-known discrepancies. We recommend that manuscripts clearly distinguish half-cell from full-cell results and discuss why RDE metrics may not translate directly to MEA performance.

Reported power densities vary widely in units (mW/cm^2^, mW/m^2^, W/cm^2^, W/m^2^) and normalization (geometric vs. electrochemically active area), creating confusion in cross-study comparisons. We recommend adopting IUPAC-recommended units and clearly specifying the basis for normalization in all performance reporting.

Addressing these methodological issues would not only improve individual studies but also accelerate the field by enabling meaningful comparison across laboratories and guiding rational catalyst design.

**Figure 8:**
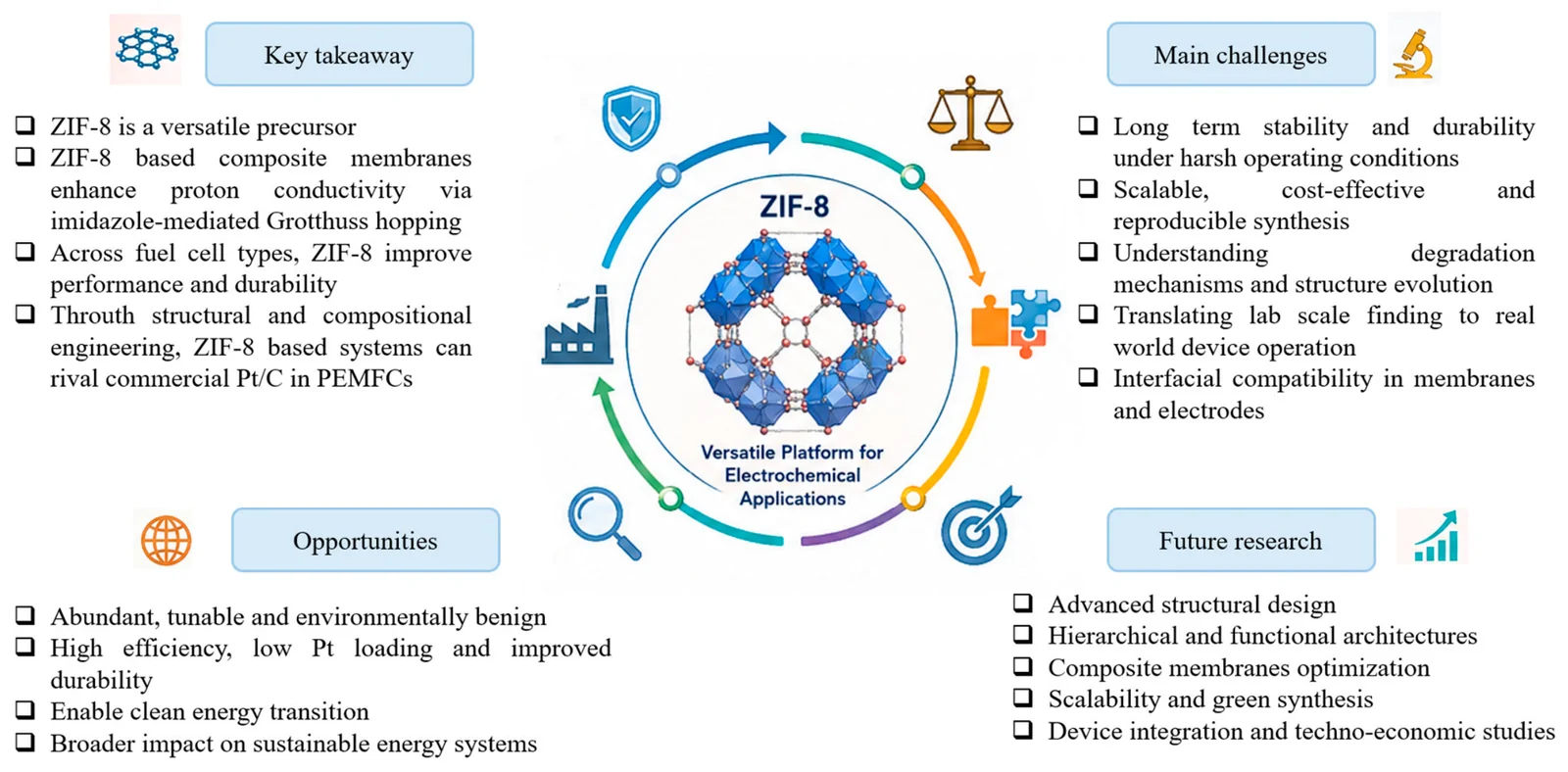
Summary of critical analysis and future perspectives based on the performance of ZIF-8 for electrochemical applications.

Moreover, despite improved progress in ZIF-8-based electrochemical technologies, several critical challenges must be addressed to enable commercial viability (Figure 8). The foremost obstacle is the durability–stability trade-off in ZIF-8-derived catalysts. While Fe–N–C catalysts demonstrate improved initial activity (P_max > 1.3 W cm^−2^), they suffer from rapid performance degradation (>80% loss within 35 h) primarily due to Fenton reactions generating reactive oxygen species that attack carbon supports and demetallate Fe–N_x_ active sites [110,111,112,113,114,115,116,117,118,119,120,121,122,123]. Advanced strategies like Zr co-doping (40% retention after 100 h) and graphitic carbon encapsulation show promise but require further optimization. This stability challenge is particularly acute in acidic PEMFC environments compared to alkaline AEMFC or neutral MFC systems, where degradation mechanisms differ significantly due to varying pH conditions and reactive oxygen species formation pathways.

Scalability and synthesis reproducibility remain significant hurdles across all applications. Most reported syntheses employ batch processes with rigorous conditions (solvothermal at 150 °C, pyrolysis at 900–1000 °C), limiting industrial translation. The development of continuous-flow synthesis, aqueous-based routes (avoiding toxic DMF), and energy-efficient Joule heating carbonization (vs. conventional furnace heating) could address these limitations while maintaining performance metrics [120,121,122,123,124,125,126,127,128,129,130,131]. It is important to note that synthesis requirements vary by application—membrane applications demand well-dispersed nanoparticles without agglomeration, while catalyst applications require precise control over pyrolysis temperature and heteroatom doping levels, necessitating application-specific optimization rather than a one-size-fits-all approach.

Device-specific challenges must be recognized and addressed separately. In PEMFCs and DMFCs, membrane degradation from radical attacks and catalyst dissolution remain critical issues. AEMFCs face additional challenges including low hydroxide conductivity and CO_2_ poisoning of anion-exchange membranes. MFCs operate under mild conditions but suffer from low power densities and biofouling of cathodes. Zinc–air batteries require efficient bifunctional catalysts for both ORR and OER, while supercapacitors demand high surface area and electrical conductivity. Each application has distinct performance metrics—PEMFCs prioritize power density and durability, DMFCs emphasize methanol crossover suppression, MFCs focus on long-term stability and cost-effectiveness, and supercapacitors require high specific capacitance and rate capability. Direct comparisons across device types are therefore scientifically invalid without appropriate normalization and contextualization.

Membrane integration faces challenges of filler agglomeration at high ZIF-8 loadings (>10 wt%), creating non-selective defects that compromise mechanical integrity and increase fuel crossover [125,126,127,128,129,130,131]. Advanced strategies such as in situ ZIF-8 growth within polymer matrices, 3D core–shell architectures (ZIF-8@PCM), and covalent crosslinking offer pathways to achieve uniform dispersion at industrially relevant loadings. However, the optimal ZIF-8 loading and dispersion strategy differ substantially between PEMFC, DMFC, and AEMFC membrane applications due to differences in membrane chemistry (Nafion vs. PBI vs. PVA) and operating conditions.

Future research directions should prioritize: (i) mechanistic understanding of degradation via operando spectroscopy and DFT calculations to guide rational design of durable catalysts, recognizing that degradation pathways differ fundamentally between acidic, alkaline, and neutral environments; (ii) development of hierarchical pore structures that balance active site density with mass transport, particularly crucial for thick electrodes in practical MEAs and for different fuel types (H_2_, methanol, wastewater); (iii) exploration of earth-abundant alternatives to Fe (e.g., Mn, Cu) with improved stability, tailored to specific application requirements; (iv) integration of ZIF-8 with emerging materials like MXenes, covalent organic frameworks, and deep eutectic solvents for synergistic effects across multiple electrochemical systems; and (v) standardization of testing protocols for each device type (RDE vs. MEA, operating conditions, durability testing) to enable fair catalyst comparison within, rather than across, application categories [122,123,124,125,126,127,128,129,130,131].

Economic viability requires reducing synthesis costs while maintaining performance. The 48-fold cost advantage of ZMG composite over Pt/C demonstrates feasibility for MFC applications, yet large-scale production of high-quality ZIF-8 (current cost ~$50–200/g) must decrease to <$10/g for commercial relevance across all applications [125,126,127,128,129,130,131]. Notably, economic considerations differ substantially between applications—PEMFCs for automotive use demand extremely low cost per watt with high durability (>5000 h), while MFCs for wastewater treatment may tolerate lower performance if capital costs are sufficiently reduced. Life-cycle assessment and techno-economic analysis should therefore be conducted on an application-specific basis to guide material selection and synthesis optimization.

A fundamental distinction must be maintained throughout future research efforts: while the materials science principles governing ZIF-8 synthesis, functionalization, and characterization are shared across applications, the performance evaluation, optimization strategies, and techno-economic considerations are inherently device-specific. This review has organized the performance analysis accordingly in Section 5, with dedicated subsections for each application type, and cautions against direct cross-device performance comparisons. This framework respects the distinct nature of each electrochemical application while leveraging the common materials foundation to accelerate progress across the field (Table 15).

The stability and durability of ZIF-8-based materials represent perhaps the most critical barrier to commercial viability across all electrochemical applications (Table 16). However, degradation mechanisms differ fundamentally depending on the operating environment (acidic, alkaline, or neutral pH), the material form (membrane vs. catalyst), and the specific device architecture. Table 16 provides a systematic comparison of these degradation pathways, typical testing protocols, and mitigation strategies across applications.

For PEMFC catalysts, the primary degradation mechanism involves Fenton reactions between Fe ions and H_2_O_2_, generating reactive oxygen species that corrode the carbon support and demetallate Fe–N_x_ active sites [9,42,45,52]. This is the most extensively documented degradation pathway, supported by XPS, XAS, and TEM post-mortem analyses. Mitigation strategies include Zr co-doping to suppress H_2_O_2_ formation [45,46], graphitic carbon encapsulation to protect active sites [62], and CNT networks to enhance corrosion resistance [53]. PEMFC membranes degrade primarily through radical attack on the polymer backbone, leading to membrane thinning and pinhole formation [22,127], which can be mitigated by incorporating radical scavengers or reinforcing fillers [37,38,39,40].

For MFCs, biofouling presents a unique challenge not encountered in other applications, as microbial attachment and biofilm formation block active sites and increase mass transport resistance [80,88]. Copper-based catalysts show promise for addressing this issue through their antibacterial properties [88]. AEMFCs face challenges related to hydroxide attack and CO_2_ carbonation [75,76], while zinc–air batteries must balance ORR and OER activity while preventing carbon corrosion at high anodic potentials [93,94,95,96].

Despite these application-specific differences, several cross-cutting challenges emerge: (i) insufficient test durations (many studies report <100 h, whereas commercial applications require >5000 h); (ii) lack of standardized testing protocols for each application; and (iii) limited understanding of degradation mechanisms at the molecular level. We recommend that future studies adopt standardized accelerated stress tests (30,000 cycles for catalysts, extended chronoamperometry for membranes) and employ operando characterization techniques to elucidate degradation pathways in real time.

While electrochemical impedance spectroscopy (EIS) and activation energy calculations provide valuable insights into proton conduction, definitive identification of transport mechanisms requires complementary advanced techniques. Table 17 summarizes the characterization methods available for distinguishing Grotthuss-type (hopping) from vehicle-type (diffusional) proton transport and for probing catalytic active sites.

Activation energy (Ea) alone is insufficient to definitively identify the proton-transport mechanism, as both Grotthuss and vehicle mechanisms can exhibit similar Ea values depending on the system and consequently, complementary techniques are needed. Firstly, isotope labeling provides the most direct evidence: if proton conduction proceeds via the Grotthuss mechanism, replacing H_2_O with D_2_O significantly reduces conductivity due to the heavier deuteron’s slower hopping rate. If the vehicle mechanism dominates, the conductivity reduction is less pronounced because diffusion of the carrier species (e.g., H_3_O^+^) is less affected [19]. Secondly, quasi-elastic neutron scattering (QENS) distinguishes localized (hopping) from long-range (diffusional) motion by analyzing the energy transfer of scattered neutrons. In ZIF-8 systems, QENS has been applied to study water dynamics within the hydrophobic pores, revealing that water molecules undergo restricted translational motion combined with fast rotational dynamics [19,116]. The combination of these motions—rotational reorientation enabling proton hopping and occasional translational jumps—is characteristic of the Grotthuss mechanism. Thirdly, solid-state NMR provides atomic-level information on proton environments and dynamics. ^1^H MAS NMR can distinguish between immobile framework protons (broad signals), mobile water protons (narrow signals), and protons involved in hydrogen bonding with imidazole nitrogen atoms [20,22]. The observation of fast exchange between imidazole N–H and water protons in ^2^H NMR spectra provides direct evidence for Grotthuss-type hopping. Fourthly, molecular dynamics (MD) simulations offer atomistic visualization of proton transport pathways. Classical MD and DFT-based ab initio MD simulations of H^+^ in ZIF-8 pores have revealed that protons preferentially migrate along hydrogen-bonded networks formed by water molecules and imidazole linkers, with activation barriers consistent with Grotthuss hopping [128,129,131]. These simulations enable quantification of the contribution of Grotthuss versus vehicle mechanisms under different hydration levels and temperatures.

For catalytic mechanisms, multiple complementary techniques are required to conclusively identify active sites and reaction pathways. Initially, X-ray absorption spectroscopy (XAS/XANES/EXAFS) provides direct evidence for the local coordination environment of metal centers. In Fe–N–C catalysts, EXAFS reveals Fe–N bond lengths of approximately 2.0–2.1 Å, confirming FeN_4_ coordination, while XANES indicates the oxidation state of Fe (typically Fe^2+^ or Fe^3+^). Operando XAS during ORR reveals structural changes during catalysis [45,103]. Then, Mössbauer spectroscopy is particularly valuable for Fe-based catalysts, as it distinguishes FeN_4_ sites (D1 and D2 sites with different isomer shifts) from Fe nanoparticles or Fe carbides. The relative proportion of D1 vs. D2 sites correlates with ORR activity, with D1 sites (higher isomer shift) showing higher intrinsic activity [42,62]. Thirdly, cyanide (CN^−^) or thiocyanate (SCN^−^) poisoning provides indirect evidence for M–N_x_ active sites, as these small molecules coordinate to metal centers and block catalytic activity. The degree of activity loss upon poisoning indicates the proportion of ORR current originating from M–N_x_ sites [86,87]. Lastly, rotating ring-disk electrode (RRDE) measurements quantify the electron transfer number (n) and H_2_O_2_ yield, distinguishing the direct 4e^−^ pathway (n ≈ 4, H_2_O_2_ < 5%) from the series 2e^−^ pathway (n ≈ 2, H_2_O_2_ > 50%). Fe–N–C catalysts typically show close to 4, confirming their ability to reduce O_2_ directly to H_2_O [78,80].

The combination of these complementary techniques provides a robust framework for understanding both proton-transport and catalytic mechanisms in ZIF-8-based electrochemical systems. Importantly, no single technique is definitive; mechanistic conclusions should be supported by multiple independent measurements.

## 7. Conclusions

This comprehensive review establishes ZIF-8 as a material platform for electrochemical energy conversion, demonstrating multifunctionality across catalytic and membrane domains. The unique combination of high surface area (1400–1800 m^2^/g), sodalite topology, nitrogen-rich imidazole linkers, and thermal stability (up to 550 °C) positions ZIF-8 as an important precursor for non-precious metal catalysts and composite membrane components. The central question addressed—how ZIF-8 and its derivatives function as both electrocatalysts and membrane components to enhance performance, durability, and economic viability—has been systematically examined through three interconnected sub-questions concerning membrane function, catalyst function, and applications across devices.

Catalytic applications have seen progress, with Fe–N–C catalysts derived from ZIF-8 achieving ORR half-wave potentials approaching 0.9 V in RDE measurements (0.5 M H_2_SO_4_) and PEMFC power densities exceeding 1.3 W cm^−2^ in H_2_-O_2_ MEA testing [42,47,62]. These values are comparable to commercial Pt/C under similar conditions. However, it is critical to distinguish between: (i) electrocatalytic activity measured in half-cells (RDE/RRDE) using acidic or alkaline electrolytes; (ii) actual performance in full cells (MEAs) with H_2_/O_2_ versus H_2_/air feeds; and (iii) results obtained under different operating temperatures, pressures, and relative humidities. For instance, catalysts achieving E_1_/_2_ of 0.88 V in alkaline RDE [77] should not be directly compared with acidic PEMFC performance without appropriate contextualization. Advanced strategies including heteroatom doping (N, S, B, P), bimetallic synergy (Fe–Co, Fe–Ni, Fe–Mn), and hierarchical porosity engineering have systematically enhanced intrinsic activity, mass transport, and durability. The identification of FeN_4_ and CoN_4_ as primary active sites through XAS, EXAFS, and XPS has guided rational catalyst design. These conclusions are supported by multiple independent studies using complementary characterization techniques and electrochemical testing (RDE, RRDE, MEA).

Membrane applications demonstrate ZIF-8’s ability to enhance proton conductivity through Grotthuss and vehicle mechanisms, achieving values up to 0.24 S cm^−1^ in Nafion composites (EIS at 80 °C, 100% RH) and 0.316 S cm^−1^ in PBI systems (at 160 °C, anhydrous) [37,38,39,40]. The molecular sieving effect (pore aperture 3.4 Å) provides methanol blocking capability, reducing fuel crossover to as low as 1.25 × 10^−8^ cm^2^ s^−1^, as confirmed by diffusion cell measurements [108]. These conclusions are supported by comprehensive membrane characterization including water uptake, swelling ratio, mechanical testing, ion exchange capacity, and acid retention measurements.

Critical challenges remain, particularly regarding long-term stability (<100 h for most PGM-free catalysts in acidic PEMFC environments), scalable synthesis, and degradation mechanism understanding. Stability enhancement strategies including Zr co-doping (40% retention after 100 h in H_2_-O_2_ PEMFC [45]) and graphitic carbon encapsulation [62] show potential but require further optimization. The translation from RDE to MEA performance remains challenging, as demonstrated by Liu et al. [42], who showed that RDE results may not predict PEMFC performance, emphasizing the importance of actual fuel cell testing under relevant operating conditions (H_2_/air, 80 °C, appropriate humidity and pressure).

Evidence-based conclusions are supported by: (i) proton conductivity measurements via EIS across temperature (25–180 °C) and RH (0–100%) ranges; (ii) ORR activity from RDE/RRDE in both acidic (0.1 M HClO_4_) and alkaline (0.1 M KOH) media; (iii) MEA performance from single-cell testing with specified H_2_/O_2_ or H_2_/air feeds; (iv) durability from accelerated stress tests and chronoamperometry (up to 30,000 cycles); (v) active site identification from XPS, XAS, and EXAFS; and (vi) mechanical integrity from stress–strain testing and swelling measurements. Direct cross-device comparisons have been avoided throughout this review, as PEMFCs, DMFCs, AEMFCs, MFCs, zinc–air batteries, and supercapacitors operate under fundamentally different conditions with distinct performance metrics. Claims of “surpassing Pt/C” are explicitly qualified with specific testing conditions (electrolyte, feed gas, temperature), and economic advantages are only cited where original studies provide quantitative cost comparisons (e.g., 48-fold cost advantage of ZMG over Pt/C for MFC applications [91]), with the understanding that such results cannot be extrapolated to other applications without comprehensive techno-economic analysis.

The convergence of computational design, advanced characterization, and innovative synthesis strategies provides clear pathways for overcoming current limitations. With continued research addressing durability, scalability, and integration challenges, ZIF-8-based technologies could contribute to the development of more cost-effective electrochemical energy conversion systems. However, achieving commercial viability requires meeting specific performance targets: >5000 h durability for automotive PEMFCs, <$10/g synthesis cost for ZIF-8 precursors, and validated MEA performance under relevant operating conditions. These targets remain to be demonstrated, and future research should prioritize systematic testing under industrially relevant conditions alongside comprehensive techno-economic and life-cycle analyses.

## Figures and Tables

**Table 1:** Search terms and Boolean strategy used for literature retrieval.

Category	Search Terms and Boolean Strategy
Material	“zeolitic imidazolate framework-8” OR “ZIF-8” OR “ZIF-8 derived” OR “ZIF-8 composite” OR “ZIF-8 membrane” OR “ZIF-8 catalyst”
Applications	(“fuel cell” OR “PEMFC” OR “DMFC” OR “AEMFC” OR “MFC” OR “microbial fuel cell”) OR (“zinc-air” OR “Zn-air” OR “battery”) OR (“supercapacitor” OR “electrochemical capacitor”) OR (“water splitting” OR “HER” OR “OER” OR “oxygen evolution” OR “hydrogen evolution”) OR (“CO_2_ reduction” OR “carbon dioxide reduction”) OR (“glucose fuel cell” OR “electrochemical sensor” OR “biosensor”)
Properties/Mechanisms	(“proton conductivity” OR “ion transport” OR “membrane conductivity”) OR (“oxygen reduction” OR “ORR” OR “catalyst activity” OR “electrocatalysis”) OR (“active site” OR “M–N–C” OR “Fe–N-C” OR “Co-N-C”) OR (“durability” OR “stability” OR “degradation”)
Exclusion	(NOT “drug delivery” OR “drug release” OR “biomedical” OR “cancer therapy” OR “antibacterial” NOT “gas adsorption” NOT “CO_2_ capture” NOT “photocatalysis” NOT “heavy metal removal”)

**Table 2:** Inclusion and exclusion criteria for study selection.

Criteria Type	Inclusion Criteria	Exclusion Criteria
Publication type	Peer-reviewed journal articles; review articles; high-quality conference proceedings with substantial data; books/book chapters providing foundational knowledge	Editorials, commentaries, opinion pieces, news articles, blog posts, preprints (not peer-reviewed), theses/dissertations, patent applications
Language	English-language publications	Non-English publications without reliable translation
Publication date	Publications from 2006 (year of first ZIF-8 synthesis report) to present; updates in the last 10–15 years prioritized, with seminal earlier works included	Publications before 2006 (pre-ZIF-8 synthesis)
Topic coverage (Primary)	Studies focusing on ZIF-8 or ZIF-8-derived materials (pristine, functionalized, composite, or pyrolyzed) for proton exchange membrane fuel cells (PEMFCs)	Studies not involving ZIF-8 or ZIF-8 derivatives; studies using other MOFs (e.g., MIL, UiO, HKUST) without direct ZIF-8 relevance
Topic coverage (Secondary)	Studies on ZIF-8-based materials for adjacent electrochemical applications (DMFCs, AEMFCs, MFCs, zinc–air batteries, supercapacitors, water splitting) where insights inform PEMFC development	Studies on other MOFs or materials for these applications without ZIF-8-specific data; studies purely on non-electrochemical applications of ZIF-8 (e.g., gas storage, adsorption, sensing, drug delivery)
Material focus	Studies examining ZIF-8 as: (i) catalyst or catalyst precursor (pristine, metal-decorated, pyrolyzed N-C or M–N–C); (ii) membrane component (filler, composite, free-standing membrane); (iii) functionalized or composite forms	Studies using ZIF-8 solely as a template without electrochemical characterization; studies where ZIF-8 is a minor component (<10% by weight or not structurally integral)
Performance metrics	Studies reporting relevant performance metrics: ORR activity (E_1_/_2_, j_k_, n), power density, proton conductivity (σ), ion exchange capacity (IEC), fuel crossover, durability/stability, single-cell or MEA performance	Studies reporting only synthesis or characterization without electrochemical performance data; theoretical/computational studies without experimental validation
Experimental quality	Studies with clearly described synthesis methods, characterization, and testing conditions; sufficient data (quantitative results) for comparative analysis	Studies with insufficient methodological detail to reproduce or critically evaluate results; studies with clearly flawed experimental design or unsubstantiated claims
Relevance to central question	Studies that contribute to understanding how ZIF-8 structure, composition, or processing affects electrochemical performance through identifiable mechanisms	Studies that only tangentially reference ZIF-8 without addressing mechanisms or performance

**Table 3:** Fundamental properties of ZIF-8 [18,19,20,21,22,23,24].

Property	Value/Description	Key Details
Crystal Structure	Zeolitic SOD topology (sodalite-type)	3D framework of Zn^2+^ ions tetrahedrally coordinated by 2-methylimidazolate linkers.
Pore Architecture	Cage diameter: 1.16 nm Aperture diameter: 0.34 nm	Large cavities connected by small, flexible windows, allowing molecular sieving.
Specific Surface Area (BET)	~1500–1870 m^2^/g	Values can vary based on synthesis/activation; often reported between 1500 and 1870 m^2^/g.
Pore Volume	~0.67 cm^3^/g	Typically reported around 0.66–0.69 cm^3^/g.
Thermal Stability	Stable up to ~400 °C in an inert atmosphere	Thermal decomposition begins significantly above 200 °C, with structural collapse reported beyond 400 °C in N_2_.
Surface Chemistry	Hydrophobic internal pore surface Often hydrophilic external surface	The internal pores show a low affinity for water (hydrophobic), but the external crystal surface can be hydrophilic, with variable water contact angles.

**Table 4:** Performance summary of ZIF-8 composite membranes for PEMFC applications.

Membrane System	ZIF-8 Loading	Proton Conductivity	Conditions	MEA Performance	Key Observations
Nanostructured ZIF-8 (80 nm) [2]	Pristine ZIF-8 (80 nm vs. micron-sized)	0.5 mS/cm (two orders higher than micron-sized)	94 °C, 98% RH	Not reported	Nanostructuring creates Zn–Im_2_ surface defects; hydrophilic sites enable water-mediated transport; Ea 1.14 eV (vs. 1.5 eV); alternative to chemical functionalization
Nafion/ZIF-8@PCM [37]	3D core–shell	0.24 S cm^−1^	80 °C, 100% RH	Not reported	1.5× vs. recast Nafion; Ea 8.02 kJ mol^−1^; 18.7% water uptake
PFSA/ZIF-8 (confined) [38]	~2 μm coating	Not reported	25–60 °C	385 mW cm^−2^ (60 °C)	3× vs. Nafion 117; ohmic resistance 0.30 Ω·cm^2^; water retention improved
PBI/ZIF-8 [39]	2.5–10 wt%	0.316 S cm^−1^	160 °C, anhydrous	Not reported	3× enhancement vs. pristine PBI; agglomeration at high loading; mechanical stability concerns
PBI/ZIF-8 [40]	1–5 wt%	0.163 S cm^−1^	170 °C, anhydrous	432 mW cm^−2^ (170 °C)	Acid retention improved

**Table 5:** Performance summary of ZIF-8-derived catalysts for PEMFC applications.

Catalyst System	Synthesis Method	ORR Performance (RDE)	PEMFC Performance	Durability/Stability	Key Findings
Fe_2_-Z8-C [42]	Solvent-free solid synthesis	E_onset_ = 0.902 V, E_1/2_ = 0.805 V	1.65 A cm^−2^ at 0.6 V; P_max_ = 1.14 W cm^−2^	98.2% retention after 10,000 s	Green synthesis; atomically dispersed Fe-N_4_ (3 wt%); high volumetric current density
Fe-BN-C-20 [43]	One-step pyrolysis with melamine, phenylboronic acid	E_1/2_ = 0.859 V (vs. Pt/C 0.853 V)	Not reported	91% retention after 36,000 s	B doping modulates electron density; synergistic Fe–N_x_ and BC_3_ sites
CZ-FeMn [44]	MWCNT/ZIF-8 + bimetallic FeMn	E1/2 0.81 V	P_max_ = 787 mW cm^−2^	10 mV loss after 10k cycles	Bimetallic synergy; high N content (5–9 at%)
Fe/Zr-N-C [45,46]	Zr doping + Fe gaseous deposition	E_1/2_ = 0.872 V	1.20 W cm^−2^ (H_2_/O_2_), 0.72 W cm^−2^ (H_2_/air)	40% retention after 100 h	Fe-N_5_-Zr-N_6_ dual sites; Zr suppresses H_2_O_2_ formation
FeN_4_/HOPC-c-1000 [47]	PS template forced molding	E_1/2_ = 0.80 V (20 mV vs. Pt/C)	P_max_ = 0.85 W cm^−2^	47% loss after 100 h	Hierarchical porosity enhances mass transfer; pyrolysis >800 °C critical
JH-120 (Joule heating) [48]	ZIF-8 carbonization at 900 °C	Limiting current −4.227 mA cm^−2^	Not reported	TOF 0.023 s^−1^	Higher graphitization, lower N content but higher intrinsic activity
55%100@30 [49]	Self-assembly dual-size architecture	E_1/2_ = 0.86 V	P_max_ = 0.75 W cm^−2^	50.6% retention after 100 h	Hierarchical porosity; dual-size prevents agglomeration
ZIF’-FA-CNT-p [50]	MWCNT + FA loading	Not reported	P_max_ = 820 mW cm^−2^	Not reported	MWCNT networks provide electron highways; FA increases surface area
CoNC@KJ600 [51]	In situ growth on KJ600 + NH_3_ pyrolysis	Not reported	P_max_ = 0.92 W cm^−2^	73% after 20 h	Improved Co–N–C P_max; KJ600 reduces HFR by 25 mΩ cm^−2^
Fe/N/C(4mlm)-OAc [52]	Carboxylate-assisted wet-impregnation	Mass activity 5.98 A g^−1^ at 0.85 V	P_max_ = 1.33 W cm^−2^ (3 mg cm^−2^)	80.3% loss in 35 h	Improved P_max for non-Pt; carboxylate increases Fe doping; stability poor
Fe-ZIF/CNT-1 [53]	Seeded growth on CNT seeds	Not reported	P_max_ = 480 mW cm^−2^	34% loss after 30 h AST	Secondary CNTs with high-density Fe–Nx sites; improved stability
CoFe-N-SiOCa [54]	Polymer-derived ceramic + ZIF-8 functionalization	E_1/2_ only 8 mV after ADT	P_max_ = mW cm^−2^, OCV 768 mV	Excellent (ΔE1/2 = 8 mV)	ZIF-8 functionalization doubles N content; acid leaching improves durability for HT-PEMFC
Pt@Fe–NC [55]	Hollow Fe–NC support + Pt deposition	Mass activity 1.34 A mgPt^−1^	Not reported	14.18% MA loss after 20k cycles	Strong Pt–FeNC interaction; DFT confirms weakened *OH adsorption
Pt/p-ZIF-8-C [56]	Cooperative template with surfactants	E_1/2_ = 0.915 V, MA 0.44 A mgPt^−1^	P_max_ = 801 mW cm^−2^ (H_2_/air)	30.4% MA loss after 20k cycles	Mesopores (5–15 nm) enhance mass transport; high surface area (1029.7 m^2^/g)
PtNiCo/NC [57]	Pyrolysis + Pt alloy deposition	Onset 1.03 V (50 mV vs. Pt/C)	P_max_ = 1067 mW cm^−2^	No degradation after 100 h	15% vs. Pt/C; synergistic Pt/Ni/Co effects
Fe-N-CNTs-H [58]	Fe-citrate functionalized ZIF-8	E_1/2_ = 0.818 V	P_max_ = 665 mW cm^−2^	Not reported	Huge-diameter CNTs (119 nm) enable full active site utilization
Meso-ZIF-NC-5 [59]	Soft-template with F-127, NaClO_4_, toluene	E_1/2_ = 0.82 V	Not reported	Not reported	Mesopore volume 0.513 cm^3^/g; hierarchical porosity enhances mass transport
C-ZIF-8(4/2) [60]	Two-step pyrolysis with AFC functionalization	Comparable to 40% Pt/C	P_max_ = 391 mW cm^−2^	12 mV shift after 10k cycles	NH_3_ pyrolysis creates mesopores; halide poisoning confirms Fe–N active sites
FeNC-1:30 [61]	Ball-milling + two-step pyrolysis	E_onset_ = 0.95 V, E_1/2_ = 0.78 V	P_max_ = 601 mW cm^−2^	Not reported	Ferrocene prevents agglomeration; NH_3_ increases N content
Nano-e-FeNC [62]	Destruction–reconstruction (8 nm units)	E_onset_~0.98 V, E_1/2_~0.88 V	48.5 mA cm^−2^ at 0.9 V_iR-free; P_max_ = 1.41 W cm^−2^	81% retention after 60 h	Meets DOE 2025 target; edge-type FeN_4_ sites; quantitative SD measurement (40 μmol g^−1^)
FeIM/ZIF-8 [63]	Ball-milling FeIM with ZIF-8, pyrolysis at 1050 °C, NH3 treatment	E_onset_ = 0.915 V vs. RHE, n = 3.83–3.93	P_max_ = 0.287 W cm^−2^	Not reported	Fe-ZIF precursor with Zn-ZIF support yields excellent ORR activity; high surface area (572 m^2^/g)
Co-NSC 200 [64]	Sulfurization of core–shell ZIF-67@ZIF-8	E_onset_ = 0.81 V, current density 5.5 mA cm^−2^	Not reported	Not reported	Sulfur doping enhances intrinsic activity; Co–Nx active sites
Co@N-CNT-HC [65]	Core–shell ZIF-67@ZIF-8 epitaxial growth-pyrolysis	E_1/2_ = 0.84 V, limiting current 4.70 mA/cm^2^	Not reported	97.8% retention after 8 h	Multi-dimensional architecture; synergistic Co NPs and N-doped carbon

**Table 6:** Performance summary of ZIF-8-based membranes for DMFC applications.

Membrane System	Synthesis Method	Proton Conductivity	Methanol Permeability	DMFC Performance	Key Findings
pSZ-11% (Zwitterion threaded ZIF-8) [66]	Self-confined growth in ZIF-8 membrane, thermal polymerization	3.62 × 10^−2^ S cm^−1^ at 75 °C	7.66 × 10^−9^ cm^2^ s^−1^	Good performance in acidic/alkaline systems	First zwitter-MOF membrane; dual ion conductivity; SBMA generates H-bond networks
Porous PES/ZIF-8 [67]	In situ growth via dead-end filtration	17.9 × 10^−3^ S cm^−1^ (85× enhancement)	3.88 × 10^−7^ cm^2^ s^−1^	6× selectivity vs. PES, 2× vs. Nafion	ZIF-8 provides proton pathways via imidazole linkers; hydrophobic domains reduce methanol crossover
IL-ZIF-8@PVDF/SPEEK [68]	In situ growth, IL modification, SPEEK filling	161.92 mS cm^−1^ at 80 °C	Excellent methanol barrier (CCD: 58.18 mA cm^−2^)	PPD: 114.86 mW cm^−2^; 93.7% after 320 h	IL improves compatibility; PVDF nanofiber network creates long-range H^+^ pathways

**Table 7:** Performance summary of ZIF-8-based catalysts for DMFC applications.

Catalyst System	Synthesis Method	MOR/ORR Performance	DMFC Performance	Key Findings
ZIF-8 thin film (oil–water interface) [69]	Self-assembly at toluene–water interface, room temp	Current density 32.7 mA cm^−2^, poisoning rate δ = 0.1%	Power 15.4 mW cm^−2^	First ZIF-8 thin film at oil–water interface; MOR activity comparable to Pt
Pt@ZIF-8 (in situ) [70]	Liquid–liquid interface synthesis	Peak current 48.10 mA cm^−2^, EASA 1084.7 m^2^/g	Max power 94.80 mW cm^−2^	In situ strategy yields Pt@ZIF-8 nanorods (230 nm × 3.8 μm); highest BET (1643 m^2^/g)
Pt-NCNT@CZIF8 [71]	PDA coating on CNTs; ZIF-8 growth; pyrolysis 700 °C; Pt deposition	ECSA 93.33 m^2^ g^−1^; MOR 4.32 mA cm^−2^	Not reported	PDA provides -OH nucleation sites; N-doping enhances Pt dispersion; improved CO tolerance
Pt/GA-CNx-ZIF-8 [72]	Hydrothermal self-assembly, pyrolysis 900 °C	ESA 89.0 m^2^ g^−1^; MOR 1.8× vs. Pt/C	Not reported	In situ ZIF-8 restructuring suppresses graphene stacking; N-doping creates defect sites
Pt/Co-NCNT-HPC [73]	Soft-template synthesis, Pt deposition	Mass activity 416.2 mA/mgPt; ECSA 49.7 m^2^/g	Not reported	Co-NCNT-HPC provides anchoring sites via Co–Nx and pyrrole-N bonds
Z8-NCF-P/EGs [74]	One-pot synthesis, PVP-assisted anchoring, pyrolysis 900 °C	E_onset_ = 0.663 V vs. SCE, JL 6.3 mA cm^−2^, E_1/2_ = 0.557 V	Methanol tolerance improved to Pt/C	PVP carbon shell protects active sites; pyridinic N and graphitic N enhance ORR

**Table 8:** Performance summary of ZIF-8-based membranes for AEMFC applications.

Membrane System	Synthesis Method	Ionic Conductivity	Water Uptake/Swelling	AEMFC Performance	Key Findings
ImPPO/3%ZIF-8 [75]	Solution casting	1.96 mS/cm; IEC 4.06 mmol/g	31% uptake; 15.1% swelling	PPD: 158.10 mW/cm^2^ (vs. 109.49 mW/cm^2^ for Fumasep)	Hydrophobic ZIF-8 reduces water uptake; alkaline stability 62% after 120 h; 3.4 Å pore blocks methanol
PVA/40.5%ZIF-8/GA [76]	Water-based synthesis + GA crosslinking	0.0204 S/cm	<1.4% swelling	PPD: 190.5 mW/cm^2^ (181% vs. PVA/GA)	GA crosslinking improves stability (0.19% loss after 168 h); selective OH^−^/water passage; blocks methanol

**Table 9:** Performance summary of ZIF-8-derived catalysts for AEMFC applications.

Catalyst System	Synthesis Method	ORR Performance (RDE)	AEMFC Performance	Durability/Stability	Key Findings
Fe_1_Co_2_–ZNT-900 [77]	Pyrolysis of ZIF-8/CNT/Fe/Co acetate at 900 °C	E_1/2_ = 0.847 V (surpasses Pt/C 0.834 V)	P_max_ = 0.171 W cm^−2^; 0.326 A cm^−2^ at 0.5 V	Not reported	Bimetallic FeCo nanoalloys provide M–N_x_ sites; micro-mesoporous structure enhances ORR kinetics
FeNSC2 [78]	Ball-milling with thiourea, NH_3_ pyrolysis at 950 °C	E_1_/_2_ 0.88 V; HO_2_^−^ < 10%	P_max_ = 162 mW cm^−2^	Excellent; 4 mV shift after 10k cycles	Synergistic N/S doping; high surface area (1260 m^2^/g); scalable ball-milling approach
Fe–N–C-1000 [79]	Pyrolysis at 1000 °C in Ar	E_1_/_2_ 0.87 V; Jlim −5.66 mA cm^−2^	P_max_ = 149 mW cm^−2^ (ADMFC: 49 mW cm^−2^ at 5 M methanol)	94% ECSA retention after 30k cycles	Improved methanol tolerance (2 M); Fe–N coordination increases with temperature; practical viability

**Table 10:** Performance summary of ZIF-8-based catalysts for MFC applications.

Catalyst System	Synthesis Method	ORR Performance (RDE)	MFC Performance	Durability/Stability	Key Findings
Pristine ZIF-8 [80]	Solvothermal synthesis	High pyridinic N (81.03%)	P_max_ = 2103.4 mW/m^2^ (1.62× Pt/C)	Stable > 110 h	First ZIF-8 as MFC catalyst; hierarchical porosity; abundant active sites
TiO_2_@ZIF-67/ZIF-8 [81]	Hydrothermal, core–shell	Enhanced redox activity	P_max_ = 341.5 mW/m^2^ (2.07× ZIF-67)	8.3 days at 413.43 mV	TiO_2_ coating enhances active sites and catalytic activity
Fe–N–C-900 [82]	Ultrasonic nucleation, pyrolysis 700–900 °C	E_onset_ = 0.91 V, E_1/2_ = 0.85 V	P_max_ = 1508 mW/m^2^	Not reported	Atomically dispersed FeN_4_; pyrolysis optimization critical
Fe/Fe_3_C@NiNC [83]	One-step pyrolysis at 900 °C	E_1/2_ = 0.87 V, E_onset_ = 0.95 V	P_max_ = 1600 mW/m^2^	120 h stability	CNTs encapsulate Fe/Fe_3_C NPs; Ni doping optimizes electronic structure
Fe(A)-NC [84]	Fe(acac)_3_ encapsulation, pyrolysis 900 °C	E_onset_ = 0.828 V, E_1/2_ = 0.690 V	P_max_ = 1917 mW/m^2^	Not reported	Fe source molecular size matching ZIF-8 cavity determines efficiency
Fe(A)-NC(L) [85]	ZIF-L aqueous synthesis, pyrolysis 900 °C	E_1/2_ = 0.709 V (PBS), 0.874 V (KOH)	P_max_ = 1949 mW/m^2^; COD 91.1%	Not reported	Eco-friendly aqueous synthesis; outperforms Pt/C in neutral/alkaline
FeCo-N-C@900 [86]	Pyrolysis with MWCNTs at 900 °C	E_1/2_ = 0.71 V, J_lim_ = −5.62 mA/cm^2^	P_max_ = 1557 mW/m^2^; COD 87.3%	91.4% retention after 10 h	Bimetallic synergy; M–N_x_ sites confirmed by cyanide poisoning
PAN–Co–ZIF-8–C–A [87]	Electrospinning + PAN, pyrolysis 900 °C	Atomically dispersed Co–N_x_	P_max_ = 888.3 mW/m^2^ (1.19× Pt/C); COD 89.8%	450–600 h stable voltage	1D nanofiber structure; practical demonstration (LED powering)
Cu-N/C@Cu-2 [88]	Two-step pyrolysis at 900 °C and 600 °C	E_1/2_ = 0.67 V (vs. Pt/C 0.70 V)	P_max_ = 581 mW/m^2^ (surpasses Pt/C 499 mW/m^2^)	700 h stable operation	Cu nanoparticles provide antibacterial activity; inhibits biofilm
CuCo-MOF@ZIF-8-2 [89]	Two-step hydrothermal + dual-solution	Enhanced electrochemical properties	P_max_ = 246.38 mW/m^2^ (2.49× ZIF-8)	365 mV over 12 days	MOF-on-MOF configuration; improved to conventional hydrothermal
Zn/DAFP-800 [90]	Template-guided structural transformation	Multi-heteroatom (N,S) doping	P_max_ = 2535 mW/m^2^; COD 92%	93.62% retention after 30 days	Hollow structure; outperforms Pt/C in power generation and treatment
ZMG (ZIF-8/MnO_2_/g-C_3_N_4_) [91]	Room temperature, no pyrolysis	EASA 2.13× Pt/C; R_ct 27.27 Ω	P_max_ = 12.38 W/m^3^ (comparable to Pt/C 12.74 W/m^3^)	86.73% after 100 cycles	48-fold cheaper than Pt/C; economically viable
Co–Zn/NC-30% [92]	In situ growth on MFC, pyrolysis 800 °C	E_0_ = 0.974 V, E_1/2_ = 0.858 V	Not reported	90% retention after 10,000 s	Hierarchical porous; uniformly dispersed Co NPs (5–10 nm); graphitic carbon encapsulation

**Table 11:** Performance summary of ZIF-8-based catalysts for zinc–air batteries.

Catalyst System	Synthesis Method	ORR Performance (RDE)	ZAB Performance	Durability/Stability	Key Findings
Fe-Fe_3_C@Fe–N–C [93]	Dual MOFs (MIL-100(Fe) + ZIF-8), thermal activation at 1000 °C	E_1/2_ = 0.88 V (alkaline), 0.79 V (acidic)	P_max_ = 0.76 W cm^−2^ in PEMFC; Discharge capacity: 8749 mAh g^−1^ (Li-O_2_)	Improved to Pt/C	Fe_3_C enhances oxygen affinity; Fe promotes *OH desorption; Fe-N_4_ active sites
Fe–Ni/CN [94]	Carbonization at 950 °C	E_1/2_ = 0.892 V (alkaline), 0.810 V (neutral)	P_max_ = 63.6 mW cm^−2^; Capacity: 700 mAh g^−1^	>120 h cycling	FeNi alloy NPs induce CNT growth; ZAB-type SPES for glucose detection (LOD 3.34 nM)
NHCP-1000 [95]	NaCl template, pyrolysis 700–1100 °C	E_1/2_ = 0.86 V	P_max_ = 272 mW cm^−2^	160 h stability	NaCl prevents N loss; promotes graphitization; high surface area (1240.8 m^2^/g)
NHPC1.3-900 [96]	NaCl-assisted exfoliation, pyrolysis 900 °C	E_1/2_ = 0.87 V (vs. Pt/C); J_L_ = 5.2 mA cm^−2^	P_max_ = 207 mW cm^−2^	Not reported	NaCl molten salt as template and intercalating agent; 3D nanosheet networks; high surface area (1302 m^2^/g)

**Table 12:** Performance summary of ZIF-8-based materials for supercapacitors.

Material System	Synthesis Method	Specific Capacitance	Rate Capability	Cycling Stability	Key Findings
Pristine ZIF-8 [97]	Solvothermal at 150 °C, 4 h	35.38 F/g at 1 A/g; 42.22 F/g at 10 mV/s	b = 0.74 (pseudocapacitive)	Not reported	Cubic phase (52 nm); rectangular plate morphology; R_ct_ = 254 Ω; R_s_ = 1.73 Ω
Ag@ZnO/C [98]	Thermal treatment of ZIF-8 + biofilm-assisted Ag synthesis	921 F g^−1^ at 1 A g^−1^	Not reported	95% retention after 9000 cycles	EAB-mediated synthesis increases surface area (68 m^2^/g vs. 13.5 m^2^/g); eco-friendly approach

**Table 13:** Performance summary of ZIF-8-based materials for water splitting (HER/OER).

Catalyst System	Synthesis Method	OER Performance	HER Performance	Overall Water Splitting	Key Findings
ZIF-8@CoFe_2_O_4_ (ZCFO-1) [99]	Room temperature synthesis	Overpotential 330 mV, Tafel 84 mV dec^−1^	Not reported	Not reported	Bimetallic synergy; ECSA 55.29 mF g^−1^; stable > 6 h
C,N-ZIF/TiFe [100]	Hydrothermal, calcination 700 °C	Overpotential 290 mV (near RuO_2_ 270 mV)	Overpotential 291 mV	Cell potential 1.64 V at 10 mA cm^−2^	Onion-ring carbon structures; C–N–Ti–O and N–Ti–O bonds; stable 14 h
Rh@NPCP [101]	Encapsulation in NPCP via microwave reduction	Not reported	η_10_: 20 mV (alkaline), 23 mV (acidic), 45 mV (neutral)	pH-universal HER	Encapsulation prevents aggregation/detachment; E_1_/_2_ 0.84 V for ORR

**Table 14:** Performance summary of ZIF-8-based materials for other electrochemical applications.

Application	Material System	Synthesis Method	Performance Metrics	Key Findings
H_2_O_2_ fuel cells and sensors [102]	ZIF-8/PVA hydrogel	Coprecipitation, freeze-drying	1.4 A/g, 89% retention; LOD 0.02 mM, sensitivity 8.6 mA mM^−1^ cm^−2^	Bifunctional platform; excellent selectivity; ~100% recovery in tap water
CO_2_ electroreduction [103]	C-AFC@ZIF-8	Surface functionalization, pyrolysis	93% CO Faradaic efficiency at −0.43 V	Isolated Fe-N_4_ sites; no Fe–Fe bonds; improved selectivity
Photo-bioelectrocatalytic fuel cells [104]	ZIF-8/CDs/BOD@algae	Biomimetic mineralization	J = 1767 μA cm^−2^; P_max_ = 131.8 μW cm^−2^; 74% degradation in 3 h	ZIF-8 enhances O_2_ diffusion; protects algae (86.8% viability); TCH tolerance
Glucose fuel cells [105]	Pt/NCZIF-8	Pyrolysis, Pt reduction	P_max_ = 0.54 μW/cm^2^; Voc = 323.8 mV; 7.83% decay after 300 cycles	3D porous support (944 m^2^/g); oxygen tolerance; 2.7 nm Pt NPs
Ethanol oxidation [106]	α-Ni(OH)_2_/ZNC@rGO	Pyrolysis, refluxing	EOR onset 0.34 V; 8.3 mA cm^−2^; 92% stability after 900 cycles	α-Ni(OH)_2_ disordered structure; rGO enhances conductivity
Smart membranes (light-switchable) [107]	Ag-DNA@ZIF-8	Solid-confined conversion	Off/On ratio 6.6 × 10^5^; 5 orders decrease under light	LSPR heating disrupts H-bond networks; fully reversible
DMFC membranes [108]	DNA@ZIF-8	Solid-confined conversion	0.17 S cm^−1^ at 75 °C; 1.25 × 10^−8^ cm^2^ s^−1^ permeability; 9.87 mW cm^−2^	Highest MOF proton conductivity; improved Nafion selectivity
Hydrogen storage [109]	GO-2@ZIF-8	Precipitation, DFT	3.44 wt% at 77.15 K, 5 MPa; 11.48% enhancement	van der Waals forces; 0.033% variation after 5 cycles; hydro-stability
Metal-free ORR [110]	NGPC-1000-10	Direct pyrolysis	E_onset_ = −0.017 V vs. Ag/AgCl; J_L_ = 4.69 mA cm^−2^; n = 3.81	Graphitic-N dominant; surface area 932 m^2^/g; hierarchical porosity
CoN_4_ ORR catalyst [111]	Co–N–C@F127	Surfactant-assisted pyrolysis	E_1_/_2_ 0.84 V; H_2_O_2_ < 2%; 0.87 W cm^−2^; 40 mV loss after 30k cycles	Highest PGM-free, Fe-free performance; CoN_2+2_ edge sites active
Metal-free ORR [112]	NCNS-10-900	KCl-assisted pyrolysis	E_1_/_2_ 0.88 V (vs. Pt/C 0.86 V); 17 mV degradation after 10k cycles	Surface area 2495 m^2^/g; KCl exfoliates carbon layers
ORR composite [113]	Co@NC/GCN-CNT	Hydrothermal, pyrolysis	E_onset_ = −0.07 V vs. Ag/AgCl; −8.5 mA cm^−2^; n = 3.96	Synergistic Co, GCN, CNT; improved stability
Bioinspired ORR [114]	NRC@ZIF-8	In situ solvothermal, calcination	E_1_/_2_ 0.91 V; 91.88% methanol tolerance	Positively charged N atoms; heterolytic O-O cleavage
Biomass-derived ORR [115]	RW@Z8(5)	In situ ZIF-8 growth, pyrolysis	E_1_/_2_ 0.720 V (88 mV vs. Pt/C); surface area 1459.27 m^2^/g	Eco-friendly; self-doping Fe/Mo/N/S from biomass

**Table 15:** Comparative critical analysis of ZIF-8-based materials for electrochemical applications.

Application	Key Performance Metrics	Supporting Evidence	Evidence Type	Strength of Evidence	Key Challenges	Future Directions
PEMFC Catalysts	ORR E_1_/_2_ ~0.86–0.89 V, P_max_ = 0.75–1.41 W cm^−2^	RDE measurements [41,42,43,44,45,46,47,48,49,50,51,52,53,54,55,56,57,58,59,60,61,62,63,64,65], MEA testing [42,47,49,62], XPS/XAS [43,45], DFT calculations [46,55]	Full-cell + Ex situ + Half-cell	Strong (multiple studies)	Degradation > 80% in 35 h [52], Fenton reactions	Zr co-doping, graphitic encapsulation, operando characterization
PEMFC Membranes	σ 0.163–0.316 S cm^−1^, Ea 8–15 kJ mol^−1^	EIS [2,37,38,39,40], water uptake, mechanical testing [39], acid retention [40]	Ex situ + Membrane	Strong (multiple studies)	Agglomeration at >10 wt%, mechanical stability [39]	3D core–shell architectures, in situ growth
DMFC Catalysts	MOR 32.7–48.1 mA cm^−2^, I_F/I_R 1.75	RDE/MOR testing [69,70,71,72,73], ECSA measurements [71], chronoamperometry	Half-cell	Moderate (limited MEA data)	CO poisoning, Pt aggregation	Pt alloy catalysts, metal-free alternatives
DMFC Membranes	σ 0.0179–0.162 S cm^−1^, Permeability 7.66 × 10^−9^ cm^2^ s^−1^	EIS [66,67,68], methanol diffusion cells, DMFC single-cell testing [68]	Ex situ + Full-cell	Moderate	Methanol crossover, swelling	Zwitterionic membranes, DNA-threaded ZIF-8
AEMFC Catalysts	E_1_/_2_ = 0.847–0.88 V, P_max_ = 149–171 mW cm^−2^	RDE in alkaline media [77,78,79], AEMFC MEA testing [77,79], XPS	Half-cell + Full-cell	Moderate	CO_2_ poisoning, stability in alkaline	Tridoping (N,S,Fe), bimetallic synergy
AEMFC Membranes	σ 1.96 mS/cm, IEC 4.06 mmol/g, 62% retention	EIS [75,76], alkaline stability testing, methanol permeability	Ex situ	Moderate	Low OH^−^ conductivity, chemical stability	Crosslinking, hydrophobic fillers
MFC Catalysts	P_max_ = 2535 mW m^−2^, COD removal 92%, 93.6% retention	MFC polarization curves [80,81,82,83,84,85,86,87,88,89,90,91,92], CV, chronoamperometry, biofouling tests	Full-cell	Strong (MFC-specific)	Low power density, biofouling, scale-up	Bio-inspired catalysts, Cu-based antibacterial
Zinc–Air Batteries	E_1_/_2_ = 0.86–0.892 V, P_max_ = 63.6–272 mW cm^−2^, 700 mAh g^−1^	ZAB discharge/charge cycling [93,94,95,96], RDE, galvanostatic tests	Full-cell + Half-cell	Moderate	OER/ORR bifunctionality, cycling stability	Salt-templating, FeNi alloy catalysts
Supercapacitors	C_s 35.4–921 F g^−1^, 95% retention after 9000 cycles	GCD, CV, EIS [97,98]	Ex situ	Moderate	Low capacitance without carbonization	Biofilm-assisted synthesis, ZnO/C composites
Water Splitting	OER 290 mV, HER 20–291 mV, 1.64 V at 10 mA cm^−2^	LSV, chronoamperometry, Tafel plots [99,100,101]	Half-cell	Moderate	Bifunctionality, long-term stability	Rh encapsulation, onion-ring carbon structures
CO_2_ Reduction	93% CO Faradaic efficiency at −0.43 V	CO_2_ electrolysis, XANES/EXAFS [103]	Half-cell	Preliminary	Selectivity, stability	Surface functionalization, Fe-N_4_ sites
Glucose Fuel Cells	P_max_ = 0.54 μW cm^−2^, 7.83% decay after 300 cycles	Glucose fuel cell testing [105]	Full-cell	Preliminary	Low power density, Pt cost	Porous carbon supports, oxygen tolerance

**Table 16:** Comparison of degradation mechanisms, stability testing protocols, and mitigation strategies across electrochemical applications.

Application	Primary Degradation Mechanisms	Typical Stability Test Protocol	Test Duration/Cycles	Key Performance Indicator	Mitigation Strategies	Supporting Evidence
PEMFC catalysts	Fenton reaction → ROS attack → carbon corrosion and Fe demetallation [9,42,45,52]	Accelerated stress test (AST) in O_2_-saturated 0.5 M H_2_SO_4_; potential cycling 0.6–1.0 V vs. RHE	10,000–30,000 cycles	E_1_/_2_ shift, mass activity retention	Zr co-doping [45,46]; graphitic carbon encapsulation [62]; CNT networks [53]	XPS, XAS, TEM post-mortem
PEMFC membranes	Radical attack (HO•) → polymer backbone cleavage; membrane thinning; pinhole formation [22,127]	Fenton’s test (3% H_2_O_2_ + Fe^2+^, 80 °C); OCV hold at 80 °C, 100% RH	24–168 h	OCV decay, conductivity loss, hydrogen crossover	CeO_2_ scavengers; crosslinking; ZIF-8 reinforcement [37,38,39,40]	FTIR, SEM, mechanical testing
DMFC catalysts	CO poisoning of Pt sites; Pt dissolution/agglomeration [69,70,71,72,73]	Chronoamperometry in 0.5 M H_2_SO_4_ + 1 M CH_3_OH at 0.6 V	1000–3000 cycles	I_F/I_R ratio, mass activity retention	Pt alloying (PtNiCo, PtRu); N-doped carbon supports [57,71]	CV, CO stripping, TEM
DMFC membranes	Methanol crossover → cathode poisoning; membrane swelling [66,67,68]	Diffusion cell with GC/FID; 24–72 h permeation	24–72 h	Methanol permeability (cm^2^ s^−1^), selectivity	ZIF-8 molecular sieving (3.4 Å); zwitterionic modification [66,108]	GC, NMR, swelling ratio
AEMFC catalysts	Carbon corrosion in alkaline media; M–N_x_ site loss; CO_2_ poisoning [77,78,79]	AST in O_2_-saturated 0.1 M KOH; potential cycling	10,000 cycles	E_1_/_2_ shift, ECSA retention	Tridoping (N,S,Fe); bimetallic synergy [78]	XPS, RDE, AEMFC testing
AEMFC membranes	Hydroxide attack → polymer degradation; CO_2_ carbonation [75,76]	Alkaline soak (2 M NaOH, 60–80 °C); conductivity monitoring	120–168 h	Conductivity retention, IEC loss	Crosslinking; hydrophobic ZIF-8 fillers [75,76]	FTIR, NMR, IEC titration
MFC catalysts	Biofouling; carbon corrosion; active site loss [80,81,82,83,84,85,86,87,88,89,90,91,92]	Continuous MFC operation with actual/synthetic wastewater	10–30 days (240–720 h)	Power density retention, COD removal	Cu nanoparticles (antibacterial) [88]; hollow structures [90]; CNT networks [86]	SEM biofilm analysis, EIS, chronoamperometry
Zinc–Air batteries	Carbon corrosion at high potentials (OER); Zn dendrite formation; electrolyte dry-out [93,94,95,96]	Galvanostatic charge–discharge cycling at 10–50 mA cm^−2^	100–500 cycles	Voltage gap (ΔE), round-trip efficiency	Bifunctional catalysts; salt-templating [95,96]	Chronopotentiometry, XRD, SEM
Supercapacitors	Carbon surface oxidation; electrolyte decomposition; electrode delamination [97,98]	Galvanostatic charge–discharge (GCD) cycling at 1–10 A g^−1^	5000–10,000 cycles	Capacitance retention, coulombic efficiency	Biofilm-assisted synthesis; ZnO/C composites [98]	EIS, CV, SEM
Water splitting	Catalyst dissolution; bubble-induced mechanical stress; carbon corrosion [99,100,101]	Chronoamperometry at 10–100 mA cm^−2^; potential cycling	10–100 h	Overpotential shift, current density retention	Rh encapsulation; onion-ring carbon; C–N–Ti–O bonds [100,101]	TEM, XPS, chronoamperometry

**Table 17:** Advanced characterization techniques for proton-transport and catalytic mechanisms in ZIF-8 systems.

Technique	Mechanism Probed	Application to ZIF-8 Systems	Key Insight Provided	Supporting Studies
Isotope labeling (H/D exchange)	Grotthuss vs. vehicle	H_2_O/D_2_O exchange in ZIF-8 pores; conductivity comparison	Lower conductivity in D_2_O indicates Grotthuss mechanism (proton hopping)	[19,20]
Solid-State NMR	Proton mobility, local structure	^1^H, ^13^C, ^15^N MAS NMR of ZIF-8 with guest molecules	Identifies hydrogen-bonding networks, imidazole protonation states, mobile vs. immobile species	[20,22]
Quasi-Elastic Neutron Scattering (QENS)	Translational vs. rotational motion	QENS of H_2_O/ZIF-8; elastic window scans	Distinguishes localized (hopping) from long-range (vehicle) diffusion; quantifies jump lengths and residence times	[19,116]
Molecular Dynamics (MD) simulations	Atomistic transport pathways	Classical DFT, ab initio MD of H^+^ in ZIF-8 pores	Visualizes proton-hopping networks; calculates activation barriers; identifies rate-limiting steps	[128,129,131]
XAS/XANES	Active site evolution under reaction conditions	Fe K-edge XANES/EXAFS during ORR	Reveals Fe–N bond length changes, oxidation state shifts, and active site dynamics during catalysis	[45,103]
Mössbauer Spectroscopy	Fe local environment, spin state	^57^Fe Mössbauer of Fe–N–C catalysts	Distinguishes FeN_4_ (D1/D2 sites) from Fe nanoparticles; quantifies Fe species distribution	[42,62]
Cyanide/SCN^−^ Poisoning	M–N_x_ active site identification	Addition of CN^−^ or SCN^−^ to electrolyte; ORR activity comparison	Confirms M–N_x_ as active sites; quantifies active site density	[86,87]
Rotating Ring-Disk Electrode (RRDE)	ORR pathway (2e^−^ vs. 4e^−^)	Collection efficiency; H_2_O_2_ yield calculation	Quantifies electron transfer number (n) and H_2_O_2_ selectivity; indicates mechanism	[78,80]

## Data Availability

No new data were created or analyzed in this study. Data sharing is not applicable to this article.

## Abbreviations

The following abbreviations are used in this manuscript:

AEMFCs	anion exchange membrane fuel cells
BET	Brunauer–Emmett–Teller
CV	cyclic voltammetry
DMFCs	direct methanol fuel cells
DSC	differential scanning calorimetry
EIS	electrochemical impedance spectroscopy
FTIR	Fourier-transform infrared spectroscopy
GCD	galvanostatic charge–discharge
Hmim	2-methylimidazolate
IEC	ion exchange capacity
LSV	linear sweep voltammetry
MFCs	microbial fuel cells
MOFs	metal–organic frameworks
MEAs	membrane electrode assemblies
ORR	oxygen reduction reaction
PBI	polybenzimidazole
PEMFCs	proton exchange membrane fuel cells
PXRD	powder X-ray diffraction
RDE	rotating disk electrode
RRDE	rotating ring-disk electrode
SEM	scanning electron microscopy
SOD	sodalite
TEM	transmission electron microscopy
TGA	thermogravimetric analysis
XAS	X-ray absorption spectroscopy
XPS	X-ray photoelectron spectroscopy
ZIF-8	zeolitic imidazolate framework-8
